# A large‐scale retrospective study in metastatic breast cancer patients using circulating tumour DNA and machine learning to predict treatment outcome and progression‐free survival

**DOI:** 10.1002/1878-0261.70015

**Published:** 2025-04-15

**Authors:** Emma J. Beddowes, Mario Ortega Duran, Solon Karapanagiotis, Alistair Martin, Meiling Gao, Riccardo Masina, Ramona Woitek, James Tanner, Fleur Tippin, Justine Kane, Jonathan Lay, Anja Brouwer, Stephen‐John Sammut, Suet‐Feung Chin, Davina Gale, Dana W. Y. Tsui, Sarah‐Jane Dawson, Nitzan Rosenfeld, Maurizio Callari, Oscar M. Rueda, Carlos Caldas

**Affiliations:** ^1^ Cancer Research UK Cambridge Institute Li Ka Shing Centre Cambridge UK; ^2^ Department of Oncology University of Cambridge UK; ^3^ CRUK Cambridge Centre and NIHR Cambridge Biomedical Research Centre University of Cambridge and Cambridge University Hospitals NHS Foundation Trust UK; ^4^ Guys and St Thomas Hospital London SE1 9RT UK; ^5^ MRC‐Biostatistics Unit University of Cambridge UK; ^6^ Department of Radiology University of Cambridge UK; ^7^ Department of Biomedical Imaging and Image‐Guided Therapy Medical University Vienna Austria; ^8^ Centre for Oncological Research (CORE) University of Antwerp Belgium; ^9^ Breast Cancer Now Toby Robins Research Centre The Institute of Cancer Research London UK; ^10^ The Royal Marsden Hospital NHS Foundation Trust London UK; ^11^ Memorial Sloan Kettering Cancer Center New York NY USA; ^12^ Peter MacCallum Cancer Centre Melbourne Australia; ^13^ Present address: Department of Clinical Biochemistry and Institute of Metabolic Science University of Cambridge Cambridge UK

**Keywords:** ctDNA, ichorCNA, machine learning, metastatic breast cancer, shallow whole genome sequencing, tumour fraction

## Abstract

Monitoring levels of circulating tumour‐derived DNA (ctDNA) provides both a noninvasive snapshot of tumour burden and also potentially clonal evolution. Here, we describe how applying a novel statistical model to serial ctDNA measurements from shallow whole genome sequencing (sWGS) in metastatic breast cancer patients produces a rapid and inexpensive predictive assessment of treatment response and progression‐free survival. A cohort of 149 patients had DNA extracted from serial plasma samples (total 1013, mean samples per patient = 6.80). Plasma DNA was assessed using sWGS and the tumour fraction in total cell‐free DNA estimated using ichorCNA. This approach was compared with ctDNA targeted sequencing and serial CA15‐3 measurements. We identified a transition point of 7% estimated tumour fraction to stratify patients into different categories of progression risk using ichorCNA estimates and a time‐dependent Cox Proportional Hazards model and validated it across different breast cancer subtypes and treatments, outperforming the alternative methods. We used the longitudinal ichorCNA values to develop a Bayesian learning model to predict subsequent treatment response with a sensitivity of 0.75 and a specificity of 0.66. In patients with metastatic breast cancer, a strategy of sWGS of ctDNA with longitudinal tracking of tumour fraction provides real‐time information on treatment response. These results encourage a prospective large‐scale clinical trial to evaluate the clinical benefit of early treatment changes based on ctDNA levels.

## Graphical abstract

There is an unmet need in metastatic breast cancer patients to monitor therapy response in real time. In this study, we show how a noninvasive and affordable strategy based on sequencing of plasma samples with longitudinal tracking of tumour fraction paired with a statistical model provides valuable information on treatment response in advance of the results of a CT scan.

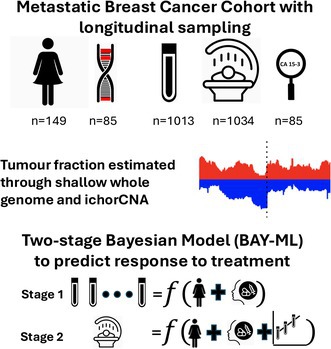

**Abbreviations**
BAY‐ML: Bayesian machine learning model
CA15‐3: carcinoma antigen 15‐3
CNAs: copy number aberrations
CT: computed tomography
CTCs: circulating tumour cells
ctDNA: circulating tumour DNA
CUH: Cambridge University Hospitals
DNA: deoxyribonucleic acid
ER: oestrogen receptor
Her2: human epidermal growth factor receptor 2
HMC: Hamiltonian Monte Carlo
NG‐TAS: next‐generation‐targeted amplicon sequencing
NUTS: no‐U‐turn sampler
OS: overall survival
PCR: polymerase chain reaction
PD: progressive disease
PFS: progression‐free survival
REMARK: reporting recommendations for tumour MARKer prognostic studies
sWGS: shallow whole genome sequencing
TM: thresholding model
TN: triple negative
VAF: variant allele frequency

## 1 Introduction

Breast cancer is the most common cancer diagnosis and the fifth leading cause of cancer death worldwide, with more than 2 million new cases diagnosed worldwide in 2022 (World Cancer Research Foundation data). Treatment options for patients with metastatic breast cancer have greatly increased, but there remains an unmet need to monitor therapy response in real time [1, 2, 3]. Accurate real‐time methods of monitoring treatment response are required to minimise time spent on ineffective therapies and improve access to more effective therapy. Recently, a clinical trial in ER+/Her2− breast cancer patients has shown some clinical utility of circulating tumour cells (CTCs) in choosing between chemotherapy and endocrine therapy [4]. CA15‐3, a tumour marker available in the clinic, is often used to monitor response but has limited sensitivity and dynamic range [4]. Additionally, we already showed in a previous study that it has inferior performance when compared with circulating tumour DNA (ctDNA) [5]. Moreover, ctDNA assays can also provide a rapid, noninvasive and dynamic way of tracking genomic evolution and detecting the emergence of resistance mutations, which could prompt therapy change [6, 7, 8].

Breast cancer genomic landscapes are dominated by chromosomal copy number aberrations (CNAs), with around 85% of tumour gene expression changes driven by these CNAs [9, 10, 11]. CNAs can be profiled using shallow whole genome sequencing (sWGS) of plasma DNA as a rapid and cheap method to characterise CNAs in ctDNA. Crucially, the detection of ctDNA in plasma using sWGS does not rely on any prior knowledge of the originating tumour genome.

Previous studies have shown the association of ctDNA and breast cancer prognosis and response to treatment. In metastatic triple‐negative breast cancer patients, a link has been identified between PFS and ichorCNA score [12]. In the neoadjuvant setting, another study has shown that the presence of residual ctDNA postoperatively was more predictive of relapse than pathological complete response [13].

Here, we evaluated the utility of ctDNA quantification using sWGS to predict treatment response in a consecutive retrospective cohort of metastatic breast cancer patients. We assessed the performance of established analysis tools to measure ctDNA levels, including ichorCNA [14], *z*‐score [15] and t‐MAD [16] and developed a Bayesian learning model that uses data from serial ctDNA measurements to dynamically predict treatment response. We also compared this approach to the use of a ctDNA targeted sequencing panel [17] and measuring CA15‐3 in the same plasma samples to determine how these different methods performed in predicting treatment response.

## 2 Materials and methods

### 2.1 The DETECT study

The study methodologies conformed to the standards set by the Declaration of Helsinki. The experiments were undertaken with the understanding and written consent of each subject. The LREC number was 07/Q0106/63. The study methodologies were approved by the local ethics committee.

### 2.2 Patient cohort and sample collection

**Fig. 1:**
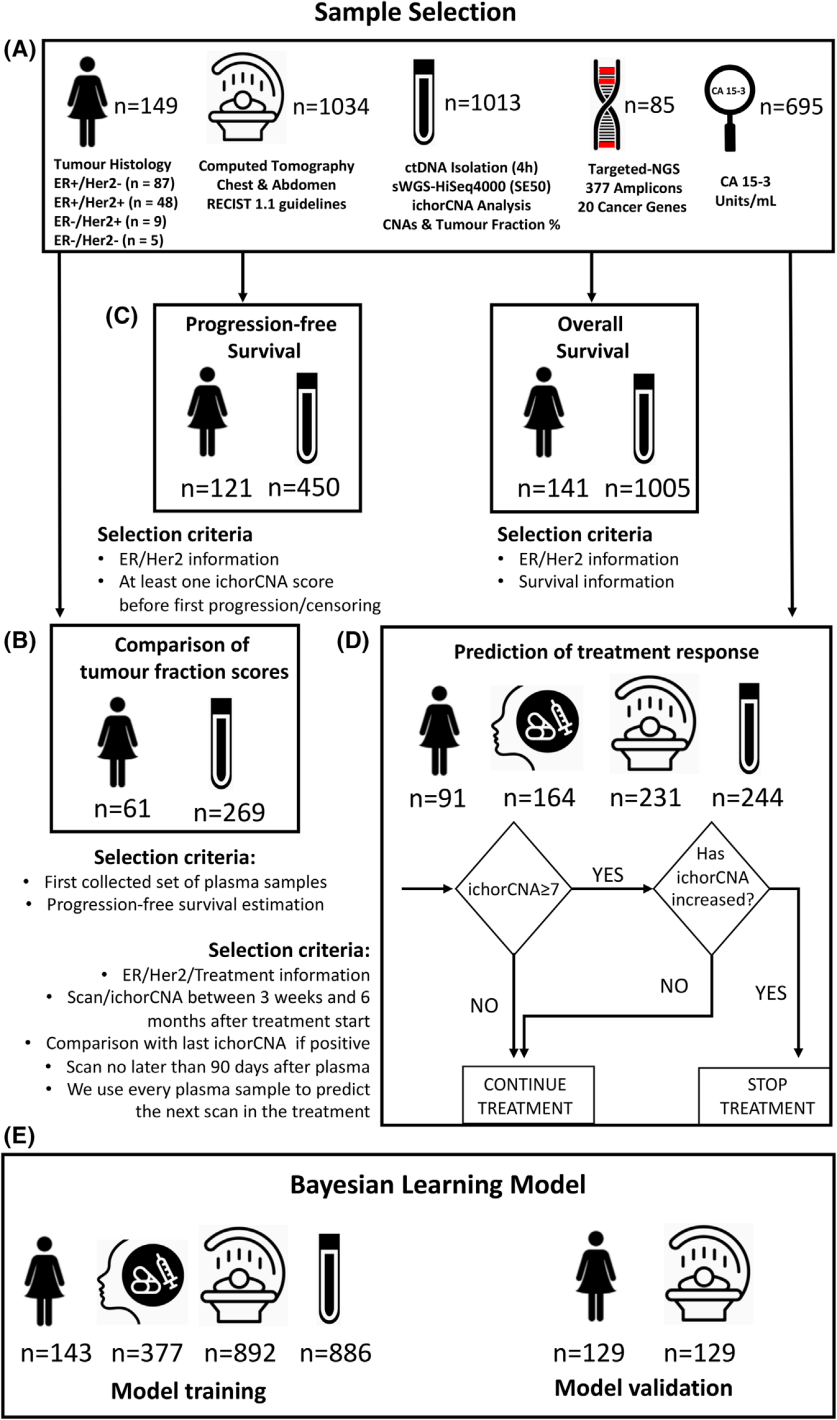
Clinical cohort and sample analysis with histology and sample selection for each analysis: (A) Patient numbers, histology and sample processing. (B) Comparison of copy number‐based tumour fraction metrics. (C) Progression free survival and overall survival analysis. (D) Prediction of treatment response using our threshold approach. (E) Bayesian learning model. CA15‐3, carcinoma antigen 15‐3; CNAs, copy number aberrations; ctDNA, circulating tumour DNA; ER, oestrogen receptor; Her2, human epidermal growth factor receptor 2; REMARK, reporting recommendations for tumour marker prognostic studies; sWGS, shallow whole genome sequencing.

A cohort of 188 patients with metastatic breast cancer was recruited into the DETECT clinical study at Cambridge University Hospitals (CUH), UK, between 2010 and 2019. Samples were collected between August 2010 and November 2019. DETECT is an ongoing investigator‐led pilot study jointly sponsored by Cambridge University Hospital NHS Foundation Trust and the University of Cambridge (IRAS number 214569). This is a retrospective translational clinical study that has been recruiting patients with metastatic breast cancer undergoing treatment at Cambridge University Hospital Trust since 2012. The study is open to any patient with metastatic breast cancer with a clinical performance status of 0–2. Blood samples are taken at regular intervals throughout treatment (typically just before a cycle of chemotherapy and upon clinic visits when on endocrine therapy). As part of the trial, buffy coats are extracted for every patient, and tumour samples (where available) can also be analysed. This study was approved by the appropriate regulatory and ethics committees (07/Q0106/63) and cosponsored by Cambridge University Hospital and the University of Cambridge. All human samples used were collected after informed consent, and the study was fully compliant with the Helsinki Declaration. Eligible patients were those women with metastatic breast cancer undergoing treatment. Serial blood samples were collected at specific time points. For patients on chemotherapy, blood samples were taken prior to the next cycle of therapy and for a minimum of four cycles. For patients on continuous treatments such as endocrine therapy, blood samples were taken at routine clinic visits (typically every 3–6 months). Cohort composition for each analysis is shown in Fig. 1, an alluvial plot is represented in Fig. S1, and the REMARK table is shown in Table 1.

**Table 1:** REMARK table.

Introduction	Main goal: Evaluate the prognostic and predictive ability of the tumour fraction score estimated by ichorCNA through shallow whole genome sequencing
Material and methods	149 breast cancer patients from DETECT with 1034 plasma samples. To derive the threshold for ichorCNA prognosis, 70 patients were used. To build the Bayesian Learning predictive model, 143 patients and 886 samples were used. The last CT scan was left out for validation (129 samples in total)
Assay methods	Shallow whole genome sequencing on single paired‐end (0.1× depth)
Study design	Prognostic model: samples from DETECT with at least one ichorCNA score before first progression/censoring split in discovery (70 patients) and validation (51 patients). End‐point: progression‐free survival. Median follow‐up of 40.3 months. Variables considered: ichorCNA (continuous and categorical). Full dataset (121 patients). End‐point: progression‐free survival. Median follow‐up of 40.3 months. Variables considered: subgroup (categorical) and ichorCNA (categorical)
	Predictive model: samples from DETECT (143 patients, 377 treatment regimes, 892 CT scans and 886 plasma samples). End‐point: result of the next CT scan. Variables considered: CT scan (binary), ichorCNA (continuous), treatment (categorical) and subgroup (categorical). The last CT scan from each available patient (129 in total) was kept out for validation
Statistical analysis methods	Prognostic model: the threshold was determined using a time‐dependent Cox model with a spline component. A segmented linear regression was applied to the fitted values to identify a suitable changepoint. This was repeated 50 times and validated with the samples held out each time
	Predictive model: two stage mixed effects Bayesian model
Results	Prognostic model: threshold of 7% for ichorCNA. For ER+HER2− patients, the hazard ratio was 4.00 [2.03, 7.88]. For HER2+ patients, the hazard ratio was 7.41 [3.37, 16.27]
	Predictive model: 75% of sensitivity and 66% specificity to detect progressive disease

### 2.3 Sample processing and analysis

In total, 1098 blood samples were collected in ethylenediaminetetraacetic acid (EDTA) tubes and processed within 1 h for plasma and buffy coat separation to prevent lymphocyte lysis and fragmentation. Samples were centrifuged at 820 ***g*** for 10 min at room temperature to separate the plasma from the peripheral blood cells. The plasma was further centrifuged at 1400 ***g*** for 10 min to remove the remaining cells and cell debris. The plasma was stored at −80 °C until DNA extraction. DNA was extracted from plasma and buffy coat. Sequencing libraries for sWGS were prepared using 5 ng of cfDNA from each sample and 50 ng of DNA from buffy coat using the ThruPLEX® Tag‐seq Kit (Takara Bio, Inc., Shiga, Japan) as described in the manufacturer's instructions. Plasma cfDNA was extracted using a minimum of 2 mL and up to 4 mL of plasma with the QIAsymphony instrument according to the manufacturer's instructions using the QIAsymphony DSP Circulating DNA extraction kit. DNA from buffy coat samples was isolated using the QIAamp DSP DNA Mini Kit (Qiagen, Manchester, UK). Extracted DNA from buffy coat was then mechanically sheared to an average length of 200 (180–220) bp target fragments using Covaris 8 microTUBE‐15 AFA Beads Strip V2 (PN 520159) tubes in a Covaris M220 22 (The Brighton Office Campus, Brighton and Hove, Brighton, UK).

The Fluidigm Access arrayTM platform (NG‐TAS) [17] was used to perform a triplicate target sequencing for 377 Amplicons to analyse 20 breast cancer genes. NG‐TAS was performed using 5 ng of cfDNA. Primers were designed with the NCBI primer‐blast tool with a Tm range of 59–61 °C. The universal primer sequences (CS1 and CS2) were added at the 5′ end of the designed primers. All primer pairs were tested alone and in multiplexed PCR reactions using 10 ng of TaqMan® Control Human Genomic DNA (Thermo Fisher Scientific, Hemel Hempstead, UK) in 10 μL reaction volumes. The coverage and performance of primers were analysed using a 2200 TapeStation instrument (Agilent, Santa Clara, CA, USA) and Hi‐seq 4000. The primers were grouped together as 7–8plex, and primers in each group were chosen to target different genes to minimise nonspecific amplification and cross‐reactivity [17].

sWGS libraries were prepared from 5 ng of cfDNA from each plasma sample or 50 ng of DNA from buffy coat using the ThruPLEX® Tag‐seq Kit (Takara Bio, Inc., Shiga, Japan) as described in the manufacturer's instructions. After barcoding PCR, all samples were analysed using 2200 TapeStation (Agilent) to measure the concentration and size of the product. If additional amplification was required, the sample was further amplified using 2–3 cycles of PCR as described with no reagents added. The additional amplification protocol is: 3 cycles of 98 °C for 20 s and 72 °C for 50 s. PCR products were pooled and cleaned with AMPure XP beads (A63880; Beckman Coulter, Brea, CA, USA) following the manufacturer's instructions. Briefly, the samples were mixed with the magnetic beads at a ratio of 1 : 1 in volume. The beads were washed twice with 80% ethanol and dried by incubating at RT for 5 min. Then, the beads were eluted with 30 μL Tris‐EDTA Buffer. The final cleaned PCR product was quantified using the qPCR KAPA Library Quantification kit (KAPA Biosystem, London, UK), and 20 nm of the eluents was submitted for sequencing on an Illumina HiSeq 4000 (Illumina, San Diego, CA, USA) to 0.1× average depth using single‐end sequencing for sWGS and > 100× for NG‐TAS.

Serum CA15‐3 levels collected as part of routine clinical care were analysed at the Cambridge University Hospitals biochemistry laboratory (accredited by the UK Accreditation Service).

### 2.4 RECIST criteria

**Fig. 2:**
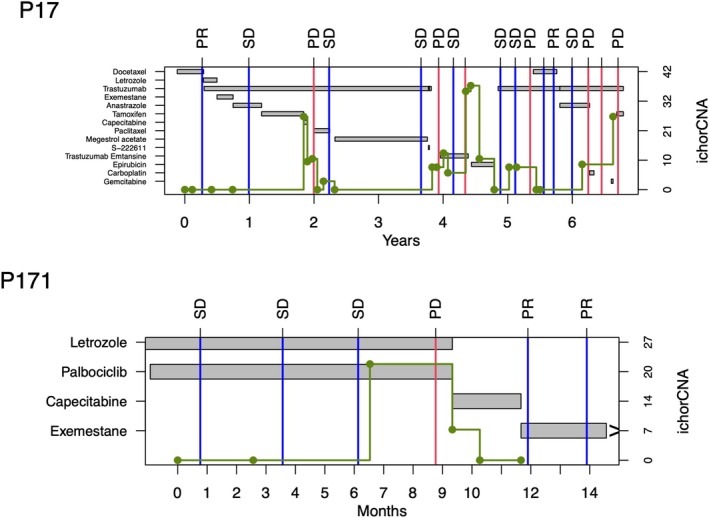
Profiles of two patients, showing the complexity of the longitudinal data available for each patient. Treatment regimens are represented with grey rectangles. CT scans are represented as vertical lines, with progressive disease (PD) in red and stable disease (SD) and partial response (PR) both in blue. Longitudinal ichorCNA scores are represented with green lines, where a circle marks the time the plasma sample was taken. Treatments ongoing at the end of follow‐up are labelled with a “>” symbol.

Progression‐free survival (PFS) for each line of treatment was calculated using RECIST 1.1 [18] guidelines as described previously (the time from the start of a line of treatment until objective progression on medical imaging using computed tomography (CT) of the chest and abdomen, or death) [19, 20]. For each line of treatment, the CT scan prior to the start of this line of treatment was used as the baseline. Imaging of the head was not included in the assessment of progression in this study. Longitudinal data available for two representative patients is shown in Fig. 2. Treatment response was also evaluated using RECIST.

#### 2.4.1 Sample selection criteria for the analyses

Only patients with at least one blood sample and one CT scan were selected for analyses (*n* = 149), with a total of 1013 blood samples and 1034 CT scans. Additional selection criteria were employed for different analyses. Figure 1 and Fig. S1 describe the sample sizes used in each analysis.

#### 2.4.2 Validation dataset

Cox proportional hazards models to predict overall survival were validated with an external dataset from the Antwerp University Hospital [21], with 80 patients (43 ER+/Her2−, 26 Her2+ and 11 ER+/Her2−) and 283 plasma samples. ichorCNA scores from sWGS were computed using the same methods used for the DETECT samples.

### 2.5 Bioinformatic and statistical analysis

#### 2.5.1 Analysis of sWGS data

Sequencing data were trimmed using trim galore and aligned using bwa‐mem to the hg19 human reference genome using bwa [22]. Next, the produced BAM files were indexed and sorted using samtools [23]. Duplicate reads were then identified and removed using picard [24]. We deemed samples with fewer than one million aligned reads as poor quality, removing any samples under this threshold at this stage.

Using the aligned data generated above, QDNAseq [25] was used to aggregate the reads into 1 Mb bins before transforming the given copy number profile into the log_2_ ratio. QDNAseq also provides GC‐correction as well as blacklisting of known problematic regions. We normalised each sample using a panel of normals approach. We took the median of each bin across all buffy coat samples (*N* = 35) to generate a reference baseline log_2_ profile, which was then subtracted from all profiles. The above pipeline produced a normalised log_2_ profile for each sample. Samples with a high level of noise, defined as the standard deviation of the profiles around the segmented mean, were filtered.

From these profiles, we derived three measures of the tumour burden (ichorCNA [14], *z*‐score [15] and t‐MAD [16]). We ran a modified version of ichorCNA. First, we ran ichorCNA with the default parameters. Note that we did not use the inbuilt wrapper of HMMcopy [26] given in the ichorCNA module for binning the aligned reads, as we wanted all tested measures to use the same log_2_ profiles as input. As output, ichorCNA provides a measure of the tumour fractions and copy number profiles, both of which include an estimation of a sub‐clone contribution. We used the following procedure to choose amongst the different solutions returned by the software:

- Compute the relative differences of the log‐likelihood of each solution and the maximum likelihood.
- Remove those solutions where that difference was larger than −0.025 as possible candidates.
- Compute the relative differences of the fraction of subclonal events of each solution and the maximum fraction of subclonal events.
- Remove those solutions where that difference was larger than 0.25.
- Pick the solution with the maximum likelihood.

tMAD requires segmentation as input, as produced by QDNASEQ (DNAcopy [27]), over the independent bin values. Note that tMAD only gives an estimate of the tumour fraction and not a copy number profile.

The *z*‐score was obtained as follows: starting from the normalised log_2_ ratio profiles, we removed all bins with a log_2_ value below 2. Next, we established the mean and standard deviation from our panel of normals (buffy coat samples) for each bin. Using these, we calculated a *z*‐score for each bin. The summary *z*‐score was taken as

$$z‐\text{score}=\sum_{c=1}^{23}\sum_{i=1}^{N_{c}}\left|Z_{c,i}\right|/\sum_{c=1}^{23}N_{c}$$

Like, tMAD, the *z*‐score method does not generate a copy number profile.

These three measures were tested on a preliminary data set (Fig. 1B).

Mutational profiling was performed using the NG‐TAS pipeline [17], with the maximum variant allele frequency (VAF) of any somatic mutation detected used to assess its predictive performance.

#### 2.5.2 Response data set

The disease status, progressive disease (PD) or not (progression: 1 = yes, 0 = no) at each CT scan, was determined according to the RECIST 1.1 criteria [18]. More specifically, the nonprogressive disease category encompassed all the patients with radiographically determined stable disease, partial or complete response. Patients were then followed during standard‐of‐care (cytotoxic) chemotherapy, targeted or endocrine therapy. Note that PD is repeatedly measured during follow‐up, that is the PD outcome for each patient is recorded at each visit, and as a result, it can switch between 0 and 1 repeatedly over time.

#### 2.5.3 Progression‐free survival analysis

We built several Cox models. First, using the preliminary dataset with fitted simple Cox models with each of the tumour burden scores as a single time‐dependent continuous variable predictor. We used the counting process notation to build the Cox models. We compared the discrimination ability of each of them through the *c*‐index [28]. Then, we selected all patients with ER/Her2 information and at least one plasma sample before progression/censoring (Fig. 1C). Next, we developed a simple threshold to discriminate low/high‐risk patients as this would facilitate clinical implementation. We hypothesised that the hazard ratio of ichorCNA would be nonlinear, but it could be represented with two different slopes. The value where the slope changes would be the threshold for low/high risk. In order to find that threshold, we randomly sampled 70 patients and fitted a Cox model with disease subtype and ichorCNA score as predictors. ichorCNA was modelled using a spline term. We then fitted a segmented linear regression to the fitted values to identify a suitable changepoint. We repeated this process 50 times and obtained a mean changepoint of 6.70%. As ichorCNA does not output decimals, we set our threshold as at least 7%. We validated this threshold using, for each random subsample, the rest of the patients not selected as a validation set, obtaining a mean hazard ratio of 1.54 and a median *c*‐index of 0.64. We also fitted models to each subtype independently and computed hazard ratios. These analyses were performed with the r packages survival [29], segmented [30] and rms [28].

We also fitted Cox models for overall survival using ichorCNA as a time‐dependent variable.

#### 2.5.4 Prediction of treatment response

For this analysis, we selected those cases where the scan and the ichorCNA plasma sample had been obtained between the first 3 weeks and the first 6 months after the treatment started. We also omitted those cases where the ichorCNA sample was taken more than 3 months before the scan (Fig. 1D). Using our stopping rule (Fig. 1D), we tested a total of 244 possible interventions over 164 treatment regimens on 91 different patients.

#### 2.5.5 Bayesian learning model (BAY‐ML)

We considered the problem of predicting the probability of disease progression at any timepoint during follow‐up based on longitudinal measurements of ctDNA. Thus, for each subject, we had a set of continuous longitudinal measurements (we did not use the threshold values derived in the previous section) and a set of longitudinal primary outcomes (a binary variable indicating whether the subject has progressed or not at the specific timepoint). The goal of the analysis was to provide, for each subject, an estimated probability of progression and a quantification of the uncertainty of this estimate. The statistical model we implemented had two components, one to model the longitudinal ctDNA measurements and one to model the longitudinal progressive disease (PD) status. The data available for each patient *i* (*i* = 1, …, *n* = 143) was

$$\begin{array}{l} {}[{\text{ctDNA}}_{i}\left(s_{i1}\right),{\text{ctDNA}}_{i}\left(s_{i2}\right),\ldots ,{\text{ctDNA}}_{i}\left(s_{im_{i}}\right),{\mathrm{PD}}_{i}\left(t_{i1}\right),\\ \;{\mathrm{PD}}_{i}\left(t_{i2}\right),\ldots ,{\mathrm{PD}}_{i}\left(t_{ik_{i}}\right),X_{i}] \end{array}$$

where ${\text{ctDNA}}_{i}\left(s_{i1}\right)$ was the value of ctDNA at time *s*_1_, ${\mathrm{PD}}_{i}\left(t_{i1}\right)$ was the progressive disease status at time *t*_1_, and **X**_*i*_ were the rest of the covariates. Note that timepoints *s* and *t* were indexed by *i* since patients were measured at different timepoints. Here, ctDNA refers to the output of the ichorCNA algorithm after being arcsine transformed [31]. The first CT scan was designated time zero and blood samples taken before these days were assigned negative time values. Also, ctDNA data collected after the last CT scan were not used. The covariates included time (years) since first CT scan, ER status (positive; negative), Her2 status (positive; negative), treatment duration (years) and treatment regime. Treatment regime was defined based on the base treatment compound or the add‐on if the base treatment was missing.

#### 2.5.6 The two‐stage approach

In the first stage, the dynamics of the repeated ctDNA measurements were summarised by random effects obtained by fitting a linear random effects model, and in the second stage, the resulting random effects were used as covariates in a logistic regression model to predict the risk of PD. Let ctDNA_*i*_(*s*_*ij*_) = ctDNA_*i*_(*s*) represent the continuous ctDNA longitudinal measurements for individual *i* at measurement time *s*_*ij*_ (*j* = 1, …, *m*_*i*_). The first stage model can be written as follows:

(1) $${\text{ctDNA}}_{i}\left(s\right)=X_{1i}\left(s\right)\beta_{1}+Z_{1i}\left(s\right)b_{i}+\varepsilon_{i}$$

where vectors **X**_1*i*_(*s*) and **Z**_1*i*_(*s*) are *p*‐ and *q*‐dimensional covariates corresponding to fixed and random effects, respectively. **X**_1*i*_(s) includes all covariates mentioned above. The vector **β**_1_ contains the fixed effect parameters. The vector **b**_*i*_ = (*b*_*i*0_, *b*_*i*1_)′ contains the random effects for patient *i*, and it is distributed as *N*(0, **∑**_*b*_). This corresponds to a random intercept and slope for subject *i*, and with

$$\sum_{b}=\left[\begin{array}{cc} \sigma_{11}^{2} & \sigma \\ \sigma & \sigma_{12}^{2} \end{array}\right]$$

we obtain a random slopes and intercepts model [13]. Finally, $\varepsilon_{i}={\left(\varepsilon_{i1},\ldots ,\varepsilon_{{\mathrm{im}}_{i}}\right)}^{\prime }$ is a vector of measurement errors with $\varepsilon_{i}∼N\left(0,\sigma_{\varepsilon }^{2}{Ι}_{m_{i}}\right)$. We further assumed that **b**_*i*_ and $\varepsilon_{i}$ are independent. In the second stage, point estimates of the subject‐specific random effects, **b**_*i*_ from stage 1 were used as predictors in a random effects logistic regression model with the disease progression as the outcome. Let PD_*i*_(*t*_*ij*_) = PD_*i*_(*t*) represent the binary measurements (progression yes/no) for individual *i* at time *j* (*j* = 1, …, *k*_*i*_). Then, the second stage model can be written as

(2) $$\text{logit}\left\{p\left({\mathrm{PD}}_{i}\left(t\right)=1\right)\right\}=X_{2i}\left(t\right)\beta_{2}+Z_{2i}\left(t\right)u_{i}+\gamma^{\prime }\hat{b_{i}}$$

where $\hat{b_{i}}$ is the predicted vector of random effects from (1), **β**_2_ and **γ** are vectors of unknown parameters, and *u*_*i*_ is a random intercept, distributed as $u_{i}\sim N\left(0,\sigma_{u}^{2}\right)$. Hence, the two models are linked through *b*_*i*_. This parameterisation postulates that patients who have a lower/higher level for the ctDNA at baseline (i.e. intercept) or who show a steeper increase/decrease in their longitudinal trajectories (i.e. slope) are more likely to experience the event. **X**_2*i*_ includes time since first CT scan, ER status, Her2 status and treatment regime. The unknown parameter vector is $\Theta ={\left(\Theta_{1}^{\prime },\Theta_{2}^{\prime }\;\right)}^{\prime }$, where $\Theta_{1}^{\prime }$ and $\Theta_{2}^{\prime }$ refer to the parameter vectors for the 1st stage and 2nd stage models, respectively. Namely, $\Theta_{1}={\left(\beta_{1},\sigma_{\varepsilon },\sum_{b}\right)}^{\prime }$ and $\Theta_{2}=\left(\beta_{2},\gamma ,\sigma_{u}^{2}\right)$.

#### 2.5.7 Bayesian inference

To infer the unknown parameter vector **Θ**, we used Bayesian inference based on Markov chain Monte Carlo (MCMC) simulations. The model was fitted as follows. First, the linear random effects model (Eqn 1) was fitted to the longitudinal ctDNA data and predicted summaries $\hat{b_{i}}$ for the random effects were computed. We used the means of the posterior distribution of $\hat{b_{i}}$ as summary measures. Then, the predicted values of the random effects were used as covariates in the generalised logistic model for PD (Eqn 2).

Fitting the two‐stage model under a Bayesian framework requires first estimating the fixed effects of the Bayesian linear random effects model as well as the means of the posterior distribution of the random effects, followed by estimating the logistic model parameters and random effects in the second stage. This requires the specification of the prior distributions for the parameters of the submodels in each stage.

We used vague priors on all elements in **Θ**. Specifically, except for the intercept terms, all other elements in **β**_1_, **β**_2_, and **γ** were *N*(0, 100). The intercept terms, as well as σ were assigned a Student‐t prior distribution with mean 0, 3 degrees‐of‐freedom, and scale 2.5 [32]. We parametrised the covariance matrix **∑**_*b*_ in terms of a correlation matrix **Ω**_*b*_ and a vector of standard deviations σ through

(3) $$\sum_{b}=\sigma^{\prime }\Omega_{b}\sigma$$

Priors were then specified for the parameters on the right‐hand side of the equation. For **Ω**_*b*_, we used the LKJ‐Correlation prior with parameter ζ = 1, i.e.

$$\Omega_{b}∼\mathrm{LKJ}\left(1\right)$$

which corresponds to a uniform density over correlation matrices of the respective dimension, that is all correlation matrices are equally likely *a priori*. Following Gelman [33] we used a half Cauchy prior with a scale parameter equal to 2 for every element of σ. We also used a half Cauchy prior for σ_*u*_ with a scale parameter equal to 2.

The posterior samples were obtained from the full conditional of each unknown parameter using Hamiltonian Monte Carlo (HMC) [34] and the No‐U‐Turn Sampler (NUTS) [35]. HMC algorithms produce samples, which are less autocorrelated than those of other samplers such as the random‐walk Metropolis algorithm. Both HMC and NUTS samplers were implemented in the probabilistic programming language Stan [36]. We used the brmsr package version 2.14.4 to implement the models [37]. To monitor Markov chain convergence, we used the trace plots and viewed the absence of apparent trends in the plot as evidence of convergence. In addition, we used the Gelman–Rubin diagnostic [38] to ensure the scale reduction $\hat{R}$ of all parameters were smaller than 1.1. After fitting the model, we obtained *M* (e.g., *M* = 10 000 after burn‐in) samples for the parameter vector **Θ**. The posterior parameter estimates for the 1st stage model and the 2nd stage model are included in Table S1.

Note that there was not enough information in the data to estimate the parameters for three treatment regimes: Exemestane/Everolimus, Lapatinib and Megestrol. The parameters corresponding to these treatments were not identifiable within the fitted models. Hence, we recommend against using the model for patients treated with these regimes.

#### 2.5.8 Assessing predictive performance

We assessed the performance of the model in predicting PD by applying it to held‐out test data corresponding to the last CT per patient (for the patients with more than 1 scan). The rest of the data was used for training. This temporal validation approach [39] matches how such a model would be used in clinical practice. All results are reported as temporal validation performance metrics, obtained by applying the trained model to the test set. We use the average of the posterior PD probability.

#### 2.5.9 Reporting recommendations for tumour marker prognostic studies (REMARK)

This study complies with the guidelines for consistent and transparent reporting [40]. The details are included in Table 1.

## 3 Results

### 3.1 ichorCNA predicts progression‐free survival in all breast cancer subtypes

Several methods for ctDNA fraction estimation using sWGS CNA data of DNA extracted from plasma have been proposed, including ichorCNA [14], *z*‐scores [15] or t‐MAD [16]. We computed those scores for the first 478 samples that were processed and compared them. Figure S2 shows the correlation of those metrics, highlighting that ichorCNA, as stated in the original paper, can not accurately estimate the value when the tumour fraction is smaller than 5%. As the agreement between methods was good (Pearson's correlations between 0.85 and 0.97), in order to identify which method performed best at estimating tumour burden in plasma, we built univariable time‐dependent Cox Proportional Hazards models for PFS (*n* = 61 patients and 269 plasma samples), using each measure (ichorCNA, t‐MAD, or *z*‐score) individually and assessing performance with the *c*‐index [28]. ichorCNA performed best (*c*‐index = 0.71, SE = 0.05), followed by t‐MAD (*c*‐index = 0.68, SE = 0.06) and *z*‐score (*c*‐index = 0.61, SE = 0.06). We focused on ichorCNA for the rest of the study.

**Fig. 3:**
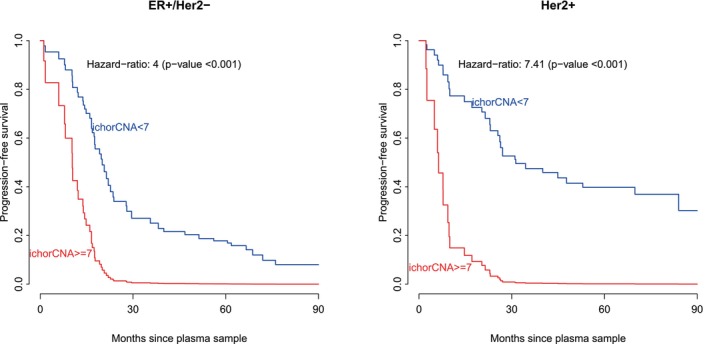
Predicted progression‐free survival curves for two patients with low and high ichorCNA scores. The hazard ratio and its test *P*‐value have been obtained from a different model fitted for each disease subtype. ER, oestrogen receptor; Her2, human epidermal growth factor receptor 2.

Having shown the prognostic ability of ichorCNA, we aimed to establish a suitable threshold to identify patients at high risk of progression in order to facilitate clinical implementation. We modeled the effect of the ichorCNA score on the hazard of progression using a spline and then fitted a segmented linear regression (Methods). We used a subsample of the patients (*n* = 70) to define the threshold and validated its prognostic ability in the rest of the patients (*n* = 51). This revealed a steady linear increase in the risk of progression followed by a changepoint and an increase of slope at a mean score of 6.70% (Fig. S3, *n* = 70). As expected, this categorisation of the score showed lower predictive power than the model using the continuous score in the subset of samples not selected in each iteration (Supplementary Methods, mean *c*‐index = 0.65 vs. 0.67, *n* = 51), though the introduction of a threshold makes clinical implementation easier. By choosing a threshold of 7%, the hazard ratio of progression for high ichorCNA score (≥ 7%) was 5.02 [3.16, 7.99] for the whole cohort (*n* = 121). When we stratified tumours by subtype, we observed differences in predictive ability. In ER+Her2− patients, the hazard ratio was 4.00 [2.03, 7.88] and the expected time until progression for patients with a ‘low risk’ ichorCNA score (< 7%) was 20.1 months, versus 10.4 months if the ichorCNA score was high. The prognostic effect was even higher for Her2+ patients, with a hazard ratio of 7.41 [3.37, 16.27] and a difference in the expected time of progression of 31.4 vs. 6.0 months. Figure 3 shows the predicted survival curves for two patients with high and low ichorCNA scores.

We observed that ichorCNA can also be used to predict overall survival. Using a continuous score with a linear term (*P* = 9.77 × 10^−8^, hazard ratio: 1.08, [1.05, 1.11], *c*‐index = 0.871, SE = 0.038) or the common threshold of 7% (*P*‐value: 0.0002, hazard ratio: 11.15, [3.16, 39.32], *c*‐index = 0.862, SE = 0.033), a higher ichorCNA value was associated with an increased risk of death. Figure S4 shows the predictions for a model using the threshold on an external dataset from the Antwerp University Hospital [21] (hazard‐ratio: 1.99, [1.20, 3.30], *c*‐index = 0.59, *P*‐value = 0.008). Overall, these results highlighted the ability of ichorCNA to predict prognosis and the utility of our proposed threshold.

### 3.2 Comparison with targeted sequencing (NG‐TAS) and CA15‐3

**Fig. 4:**
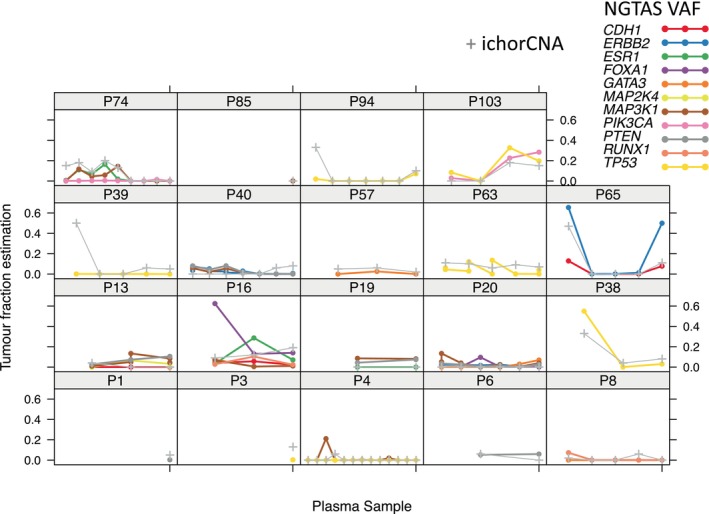
Comparison of ichorCNA scores and NG‐TAS scores for a subset of 19 patients. NG‐TAS, next generation‐targeted amplicon sequencing; VAF, variant allele frequency.

Targeted mutational sequencing data [17] were also available for 85 samples obtained from 19 patients. Figure 4 shows a good agreement between ichorCNA and NG‐TAS scores, although there are some discrepancies: cases where no mutations were identified while ichorCNA scores were high (Patients P39 and P94; possibly because there were no mutations in the subset of genes targeted by the panel) and instances where NG‐TAS showed high VAF while ichorCNA showed very low scores (first sample of P16 and last sample of P65). In this smaller sub‐cohort and using the maximum VAF observed in the samples, we did not observe a significant effect on the hazard of progression (*P* = 0.209, *n* = 45) while the ichorCNA remained significant (*P* = 0.048, *n* = 45). Combining the two scores into the same model did not improve the fit (*P* = 0.489). Although the sample sizes were very small, these results showed us that, although there are cases where NG‐TAS can be more sensitive, ichorCNA uses information from the whole genome and can be more informative.

We also compared ichorCNA and CA15‐3 scores from a sub‐cohort of 66 patients, where 695 CA15‐3 measurements were available. Figure S5 shows the trajectories of these scores in each patient. Acknowledging the limitations of comparing both datasets (different number of patients and different sampling intervals, with the CA15‐3 and the ichorCNA not always taken on the same day), we interpolated the scores across all timepoints and compared the Pearson correlations for each patient, obtaining an average of 0.13 but a range of −1 and 0.98, depending on how different the sampling times were. A Cox Proportional Hazards model using CA15‐3 to predict progression‐free survival showed lower performance in terms of *c*‐index (0.605, SE = 0.083, *n* = 38 patients) than a similar model using ichorCNA scores (*c*‐index = 0.670, SE = 0.061, *n* = 38). Moreover, CA15‐3 also predicted overall survival with a similar ability to ichorCNA (square root of CA15‐3, *P* = 0.0020, hazard ratio: 1.053 [1.02, 1.09] 95% confidence interval, *c*‐index = 0.861, SE = 0.047 while a model for ichorCNA with the same patients produced a *c*‐index of 0.87).

Considering a threshold of 2.5% VAF for NG‐TAS and 31 U·mL^−1^ (positivity threshold) for CA15‐3, there was a 68% concordance between NG‐TAS and ichorCNA, and a 60% concordance between ichorCNA and CA15‐3. Figure S6 shows the instances where these measures showed discrepant results.

### 3.3 ichorCNA ctDNA fraction predicts treatment response

Using the ichorCNA 7% threshold, we evaluated its ability to predict subsequent responses or resistance to treatment at each plasma sample time point using the results of the next CT scan. We used the following rules: (a) for a prediction of treatment response, ichorCNA < 7% or ≥ 7% if a decrease from the previous time point, (b) for a prediction of progression, ichorCNA ≥ 7% and an increase (or no change) from a previous time point (when available). The preceding time points needed to be on the same treatment to be relevant for decision‐making. The application of these rules produced a sensitivity of 43% and a specificity of 86%. The median time prior to prediction of progression for concordant decisions (stopping treatment) was 35 days (versus 29 days for discordant). For concordant decisions about continuing treatment, the median time prior to the CT scan was 40 days (versus 36 days for discordant). These differences in the time when the decisions were made were not statistically significant (*t*‐test) and would not explain the difference in predictive ability.

### 3.4 A Bayesian machine learning model (BAY‐ML) to predict treatment response

**Fig. 5:**
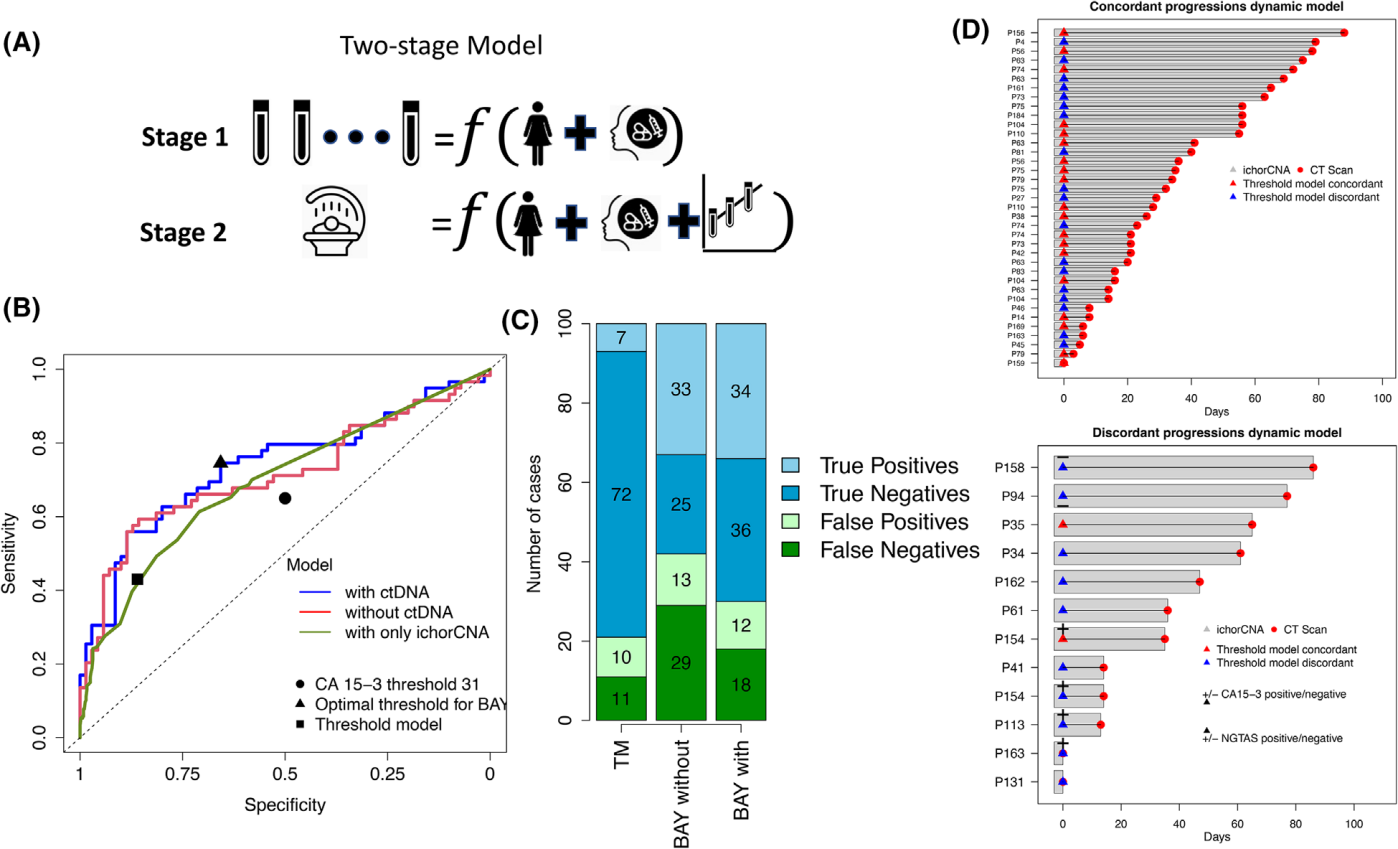
BAY‐ML model. (A) Visual summary of the two‐stage model. In the first stage, the longitudinal set of tumour fraction scores is modelled using the tumour and treatment features using a random effects model. In the second stage, the CT scan is predicted using the tumour fraction trend in the patient from the random effects and the tumour and treatment features. (B) Receiver operator characteristic (ROC) curve of the dynamic predictive model described in the text (ROC = 0.74 for BAY‐ML with ctDNA info, ROC = 0.71 for BAY‐ML without ctDNA info), together with a simple model that only uses the most recent ichorCNA value. Note that the curves have been built using the maximum number of validation samples for each method. In the case of BAY‐ML models, the predictions were obtained with the last CT scan for each patient left out when the model was fit (129 CT scans). In the case of the model with only ichorCNA, 689 CT scans. The square represents our optimal threshold for 66% specificity and 75% sensitivity, using cross‐validation. The circle represents the performance of CA 15‐3 using the recommended threshold on (C) Number of true/false positives and negatives over 100 patients when the simplest threshold model (TM in the legend) and when the longitudinal ctDNA scores are considered or not into the BAY‐ML model (BAY without and BAY with in the legend). (D) Instances where the model correctly predicted progression and instances where it did not, comparing the available information at that moment. Only predictions within 90 days of the CT‐scan and where predictions are possible in both the threshold and BAY‐ML are shown. BAY‐ML, Bayesian machine learning model; CT, computed tomography; ctDNA, circulating tumour DNA; NGTAS, next generation‐targeted amplicon sequencing; TM, thresholding model; CA15‐3, carcinoma antigen 15‐3.

Motivated by these findings, we developed a novel statistical model to predict treatment response (based on RECIST criteria) using the full history of ichorCNA scores and CT imaging (Fig. 5A). The model comprises two components, one that includes the characteristics common to the cohort (ER/Her2 status and treatment regime) and another that models the patient‐specific longitudinal ctDNA scores and disease progression measurements on CT. The model was fitted using a two‐stage approach: in the first stage, the evolution of the repeated ctDNA measurements was summarised by random effects obtained by fitting a linear mixed effects model, and in the second stage, the resulting random effects were used as covariates in a logistic regression to predict the risk of progression. Both steps included a set of independent variables, such as the treatment regime and the tumour (Supplementary Methods). The model adapts to each patient, learning from common cohorts effects such as the current treatment or the tumour subtype in both the ichorCNA trajectories and the probability of progression, but the model also learns from specific features of the patient. Leaving the final observation out for each patient (see Supplementary Methods for details), we evaluated the sensitivity for predicting progressive disease when using ctDNA information at several clinically relevant specificity thresholds. Leaving the last observation out in the model estimation, at 66% specificity, the sensitivity for detecting progressive disease was 75%, significantly higher than our previous model based on a simple stopping rule. Figure 5B shows the receiver operator characteristic (ROC) curve for the model, together with other baselines, highlighting the improvement in predictive performance, particularly in sensitivity, when longitudinal ichorCNA data is included. At the same 86% specificity, BAY‐ML outperforms the static model with 56% sensitivity (13% increase) and at the same 43% sensitivity, BAY‐ML outperforms the static model with 91% specificity (5% increase). Figure 5C shows how many patients would switch or continue treatment using a hypothetical cohort of 100 patients, while Fig. 5D shows the cases where the model did not produce concordant predictions with the CT scan, together with the information provided by ichorCNA, NG‐TAS or CA15‐3 in those cases.

### 3.5 Prediction performance according to treatment, subtype and number of plasma samples

**Fig. 6:**
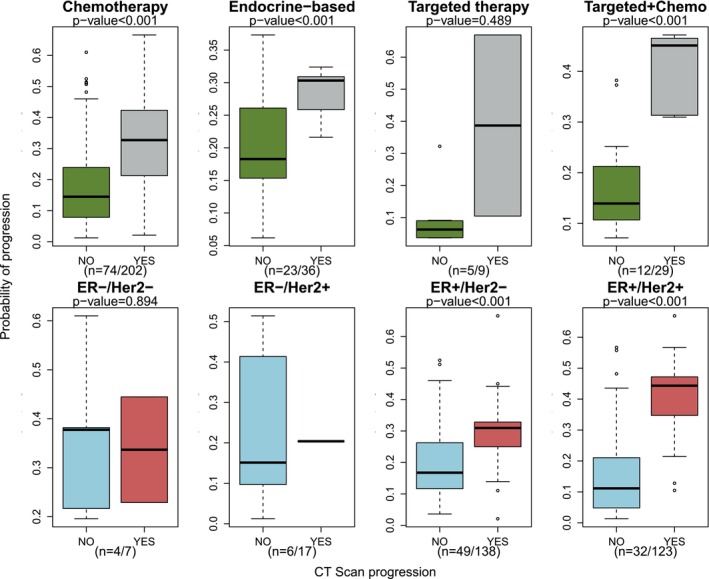
Boxplots showing the association between the probability of predicting progressive disease and the result of the CT scan depending on different treatment types (top) and on different subtypes (bottom). The number of patients and the number of predictions is indicated in each plot. *P*‐values were computed using a two‐sided *t*‐test. There were not enough observations to test the association in the ER−/Her2+ subgroup. Box plots were computed using the median of the observations (centre line). The first and third quartiles are shown as boxes, and the whiskers extend to the ±1.58 interquartile range divided by the square root of the sample size. Outliers are shown as dots. ER, oestrogen receptor; Her2, human epidermal growth factor receptor 2.

We looked at the predictive capability of ichorCNA for different treatment types (Fig. 6). Predictions of response to targeted therapy (mainly CDK4/6 inhibitors and anti‐Her2 therapy) chemotherapy and targeted + chemotherapy (Herceptin + Emtansine) showed a higher concordance with CT results than endocrine treatment alone. The predictions of progression for actual progressions were significantly higher than for no progressions, except for the case of targeted treatment due to small sample size. Blood test sampling was also less frequent for patients on endocrine treatment alone, which could also explain this apparent difference.

The bottom panel of Fig. 6 also shows that the model performed better in ER+ patients, in both Her2− and Her2+ groups; however, the sample size for ER− patients was too small to draw any conclusions.

We also looked at the ability in performance according to the available number of plasma samples. Figure S7 shows the BAY‐ML predicted probabilities compared to the CT responses stratified by the number of ichorCNA measures. The performance of the model does not seem to depend on the number of samples, and it is able to discriminate even with only three samples.

### 3.6 Discordant result analysis

In order to understand better the limitations of ichorCNA, we evaluated decisions whereby the ichorCNA values did not agree with disease status as measured using RECIST 1.1.

The ichorCNA score was high (predictive of treatment failure) but the next CT showed RECIST stable disease in 24 instances. In 11/24 cases (46%), progressive disease was observed on the subsequent CT scan, suggesting early detection of progression by ctDNA, with a median lead time of 4.8 months. In 2/24 cases, disease progression was observed in the brain (not evaluated as part of the RECIST criteria for this study).

In the cases where progression occurred on a CT scan but was not detected by ichorCNA (22 instances in total), in 5 of these cases, this followed treatment with palliative radiotherapy, which we hypothesise may have led to a reduction in ctDNA levels, separate from other systemic treatments. In 2/22 cases, a mixed response was seen, and it may be that having additional information from ctDNA could have aided the decision‐making process. For an additional 6/22 cases, low volume changes were seen (< 10 mm in < 2 lesions) which may have had limited clinical impact. In 60% of these cases, clinical management continued unchanged.

## 4 Discussion

We have demonstrated the real‐world performance of ichorCNA in predicting treatment response for metastatic breast cancer patients and have used a novel Bayesian machine learning approach, which uses longitudinal data, improving its predictive capability. This approach has advantages over other methodologies. First, all data are useful (including ichorCNA values of zero) as sWGS evaluates CNAs across the whole genome, having an important advantage over targeted sequencing, where a lack of somatic mutations in ctDNA only means that those specific mutations have not been detected. Second, the average turnaround time, including library preparation and sequencing, was less than 1 week. Third, sWGS was cheaper than currently available alternatives, with a typical cost of £100 per sample, including processing costs. This compares favorably to commercially available ctDNA tests, which provide results with a 2 weeks turnaround time, twice as long as sWGS. In addition, ctDNA monitoring using sWGS could reduce the need and frequency of CT scanning if ctDNA levels continued to be low. However, we must note that commercial and research costs are different, and costs of implementation and training must also be taken into account.

Altogether, this shows that the method we describe in this study can be cheaply deployed within the clinic for therapy monitoring in real time. Importantly, the prediction capability was agnostic of breast cancer subtype and treatment regimen, though it appears predicting response to targeted therapy and chemotherapy may be slightly more reliable than for endocrine treatment. This may reflect the biology of the disease as patients on targeted treatment and chemotherapy are likely to have more aggressive disease or have a higher tumour burden, potentially making ctDNA levels higher and more dynamic. Moreover, cytostatic treatments are probably less likely to cause large changes in ctDNA compared with cytotoxic treatments. We have also observed better performance in ER+ patients, both Her2 positive and negative, although our sample size for ER− patients was too small to be conclusive. Moreover, these differences are incorporated into the model as subtype/treatment specific parameters. For a subset of cases, we also had data on CA15‐3 levels and somatic mutations from a targeted sequencing panel. From this analysis, ichorCNA was more accurate than CA15‐3, and targeted sequencing data did not improve the predictive power, with the caveat that these comparisons were performed in a smaller number of patients. However, it is likely that a panel specifically designed with existing mutations in each tumour and sequenced at high depth can improve sensitivity to detect small tumour fractions.

Due to lower patient numbers, we were less able to comment on any specifics for triple negative breast cancer. However, previous work has already identified the prognostic value of ichorCNA in metastatic breast cancer [12], we do not believe there would be any significant difference in our model's performance for this subtype, though the optimal threshold may be different.

Our study also highlights the potential benefits of using machine learning to improve predictive power. We initially developed a simple stopping rule that is easy to implement in the clinic that is prognostic of progression, and can predict treatment response with moderate success. We improved on this using a dynamic model that uses the history of the patient to predict more accurately the probability of progression under a given treatment. As the model needs to learn patient‐specific baselines to make predictions of disease progression, for clinical implementation it would be necessary to get a couple of plasma samples from each patient before making predictions based on ctDNA.

Thinking more broadly about estimating ctDNA, it could be that this is a better marker of overall disease activity than purely measuring disease on a CT scan, as shown in a previous study [13]. We hypothesise that using the approach we describe here could provide benefits in terms of both quality of life, by reducing unnecessary toxicity, and increased access to more efficacious treatments in a timely fashion. This is also a realistic prospect, as genomic assessment using ctDNA is rapid and relatively inexpensive, making it accessible to public health systems.

## 5 Conclusions

Our work has shown that, in patients with metastatic breast cancer, a strategy of monitoring tumour fraction with longitudinal sampling of sWGS of ctDNA paired with a Bayesian statistical model provides, with turnaround times lower than 5 days, real‐time information on treatment response that can predict in advance the results of CT scans. Our findings need to be fully evaluated in a prospective randomised clinical trial to assess the use of ctDNA to make treatment decisions, comparing it also to endpoints such as quality of life and overall survival.

## Conflict of interest

NR is cofounder and officer of Inivata Ltd. Inivata had no role in the conceptualisation or design of the clinical study, statistical analysis or decision to publish the manuscript. CC is a member of AstraZeneca's iMED External Science Panel and Illumina's Scientific Advisory Board and a recipient of research grants (administered by the University of Cambridge) from Genentech, Roche, AstraZeneca and Servier.

## Author contributions

EJB, OMR, MO‐D, SK and CC contributed to conceptualisation. EJB, MO‐D, MG, S‐FC, S‐JS, SK, S‐JD, MC, AM, RM, RW, DWYT, DG and OMR contributed to methodology. EJB, MO‐D, MG, S‐FC, S‐JS, SK, AM, JL, JK, RM, S‐JS, NR, OMR, AB, FT, JT and RW contributed to investigation. EJB, MO‐D, SK, OMR and CC contributed to visualisation. CC and EJB contributed to funding acquisition. EJB and JK contributed to project administration. CC contributed to supervision. EJB, MO‐D, SK, AM, RW, OMR and CC contributed to writing—original draft. All authors read and contributed to the final manuscript.

## Supporting information


**Fig. S1.** Alluvial plot showing selection of plasma samples for each analysis in the study.
**Fig. S2.** Comparison of different scores to measure tumour fraction in ctDNA.
**Fig. S3.** Identification of a threshold for the ichorCNA score using a spline term and segmented linear regression.
**Fig. S4.** Overall survival in DETECT and Antwerp data.
**Fig. S5.** Comparison of CA15‐3 and ichorCNA scores to estimate tumour fraction in 66 patients.
**Fig. S6.** Discrepant results of ichorCNA measured with sWGS, mutant VAF measured with NGTAS and CA15‐3.
**Fig. S7.** Prediction probabilities of progressive disease produced by BAY‐ML compared to the outcome of the CT scan, stratified according to the number of plasma samples available.

**Table S1.** Parameter estimates in BAY‐ML.

**Table S2.** Treatment information for the patients.

**Table S3.** RECIST scores for the patients.

**Table S4.** ichorCNA scores for the patients.

**Table S5.** VAF for the NGTAS panel.

**Table S6.** CA15‐3 scores for the patients.

**Table S7.** Comparison of tumour fraction scores.

## Data Availability

Tables S2–S7 contain the processed data used in this study (Table S2 contains the clinical data, Table S3 contains the CT scan results, Table S4 contains the ichorCNA values, Table S5 contains the NGTAS data, Table S6 contains the CA15‐3 values and Table S7 contains the different methods compared to estimate tumour fraction. All times are considered as the difference in days from the date of the first scan of the study). The raw sequencing files have been deposited at the European Genome‐Phenome Archive, EGAS50000000911 (http://www.ebi.ac.uk/ega/), which is hosted by the European Bioinformatics Institute, under study name “Shallow whole genome sequencing of ctDNA samples from DETECT study”. Code is available at our github repository (https://github.com/Rueda‐Lab/BAY‐ML).
