# Supplementary material for: A large‐scale retrospective study in metastatic breast cancer patients using circulating tumour DNA and machine learning to predict treatment outcome and progression‐free survival
Source: Mol Oncol. 2025 Apr 15;19(12):3518–34. doi: 10.1002/1878-0261.70015 (PMC12688166; doi:10.1002/1878-0261.70015)
Supplement: Supplementary file 1 — Fig. S1. Alluvial plot showing selection of plasma samples for each analysis in the study. Fig. S2. Comparison of different scores to measure tumour fraction in ctDNA. Fig. S3. Identification of a threshold for the ichorCNA score using a spline term and segmented linear regression. Fig. S4. Overall survival in DETECT and Antwerp data. Fig. S5. Comparison of CA15‐3 and ichorCNA scores to estimate tumour fraction in 66 patients. Fig. S6. Discrepant results of ichorCNA measured with sWGS, mutant VAF measured with NGTAS and CA15‐3. Fig. S7. Prediction probabilities of progressive disease produced by BAY‐ML compared to the outcome of the CT scan, stratified according to the number of plasma samples available. [file MOL2-19-3518-s007.zip › FiguresS1-S7_Legends.docx]

Supplementary Figure 1. Alluvial plot showing selection of plasma samples for each analysis in the study. BAY-ML: BAYesian Machine Learning model. ER: oEstrogen Receptor. Her2: Human epidermal growth factor receptor 2. NGTAS: Next Generation-Targeted Amplicon Sequencing. OS: Overall Survival. PFS: Progression-Free Survival

Supplementary Figure 2. Comparison of different scores to measure tumour fraction in ctDNA. Lower panels show scatterplots of each pair of measures with a smoothed regression line in red. Diagonal panels show a histogram of each score. Upper panel shows the Pearson correlation (r) for each pair of scores.

Supplementary Figure 3. Identification of a threshold for the ichorCNA score using a spline term and segmented linear regression. A) 50 subsamples (n=70) to identify a threshold for the ichorCNA values based on the changepoint in the slope of the log relative hazard. B) c-indexes obtained with the threshold on the subset of patients (n=51) not chosen in each iteration. C) p-values for the hazard ratio obtained with the threshold on the subset of patients (n=51) not chosen in each iteration. D) Illustration of the threshold selection method based on the slope of the log relative hazard changes, using the whole cohort (n=121). E) Illustration of the threshold selection method based on the slope of the log relative hazard changes, using the ER+/Her2- cohort (n=67). F) Same for the Her2+ cohort (n=49). The application of a similar strategy to each subtype independently suggests a higher threshold for ER+ve/Her2-ve patients and a slightly lower threshold for Her2+ve patients (both ER+ve and -ve). The number of triple negative patents was too small for this stratified analysis.

Supplementary Figure 4. Overall Survival in DETECT and Antwerp data. A) Overall survival predicted curves for groups of patients with low and high ichorCNA scores based on our 7% threshold. As the number of samples in some of the groups is small, the model assumes that the hazard ratio of ichorCNA is the same in each subtype (hazard-ratio: 11.15, 95% confidence interval [3.16, 39.32], p-value < 0.001) B) Same for the validation cohort (hazard-ratio: 1.99, 95% confidence interval [1.20, 3.30], p-value= 0.008). We note the difference in baseline survival between both cohorts, due in part to discrepancies in follow-up. ER: oEstrogen Receptor. Her2: Human epidermal growth factor receptor 2

Supplementary Figure 5. Comparison of CA15-3 and ichorCNA scores to estimate tumour fraction in 66 patients. CA15-3: Carcinoma Antigen 15-3.

Supplementary Figure 6. Discrepant results of ichorCNA measured with sWGS, mutant VAF measured with NGTAS and CA15-3. 96 instances where the CT scans were done less than 90 days from or to the plasma sample and the CA15-3 was taken 15 days apart from the plasma sample measure are considered, Discrepancies are considered based on the 7% or greater threshold for ichorCNA, 30 for CA15-3 and 2.5% for VAF. Circles represent results of the CT scans (blue stable or response and red progressive disease). Upper triangles represent positive results of ichorCNA and lower triangles represent negative results. The colour of the triangles represents the result of NGTAS on the same plasma sample (grey indicates no NGTAS, red positive and blue negative). + represent positive CA15-3 tests and – negative results. CA15-3: Carcinoma Antigen 15-3. Dis: Disease. ER: oEstrogen Receptor. Her2: Human epidermal growth factor receptor 2. NGTAS: Next Generation-Targeted Amplicon Sequencing. Resp: Response.

Supplementary Figure 7. Prediction probabilities of progressive disease produced by BAY-ML compared to the outcome of the CT scan, stratified according to the number of plasma samples available. Box plots were computed using the median of the observations (centre line). The first and third quartiles are shown as boxes, and the whiskers extend to the ±1.58 interquartile range divided by the square root of the sample size. Outliers are shown as dots. CT: Computed Tomography.
