# Supplementary figures and images for: A large‐scale retrospective study in metastatic breast cancer patients using circulating tumour DNA and machine learning to predict treatment outcome and progression‐free survival
Source: Mol Oncol. 2025 Apr 15;19(12):3518–34. doi: 10.1002/1878-0261.70015 (PMC12688166; doi:10.1002/1878-0261.70015)

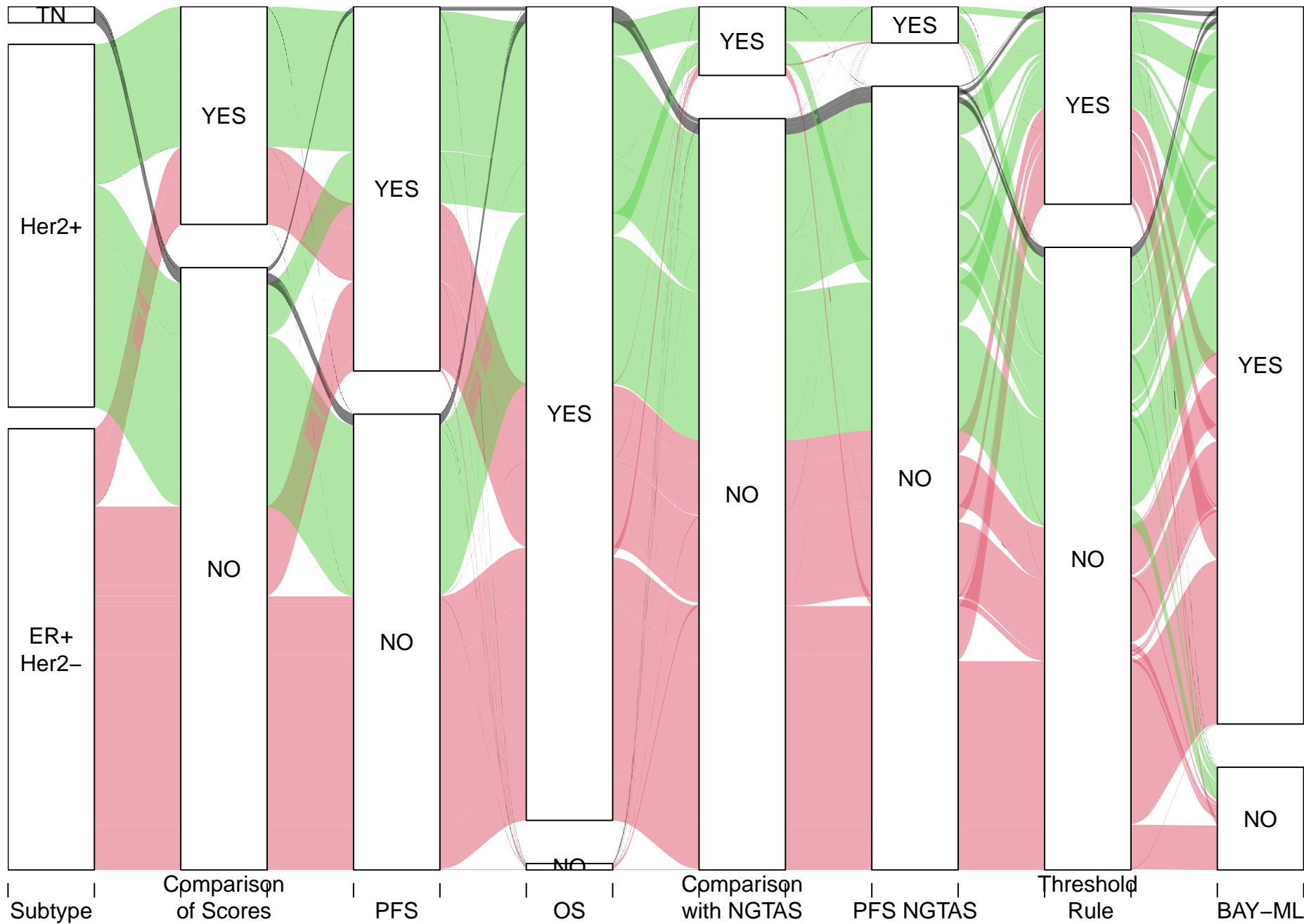

Supplement: Supplementary file 1 — Fig. S1. Alluvial plot showing selection of plasma samples for each analysis in the study. Fig. S2. Comparison of different scores to measure tumour fraction in ctDNA. Fig. S3. Identification of a threshold for the ichorCNA score using a spline term and segmented linear regression. Fig. S4. Overall survival in DETECT and Antwerp data. Fig. S5. Comparison of CA15‐3 and ichorCNA scores to estimate tumour fraction in 66 patients. Fig. S6. Discrepant results of ichorCNA measured with sWGS, mutant VAF measured with NGTAS and CA15‐3. Fig. S7. Prediction probabilities of progressive disease produced by BAY‐ML compared to the outcome of the CT scan, stratified according to the number of plasma samples available. [file MOL2-19-3518-s007.zip › FigureS1.pdf]

1 2 3 4 5

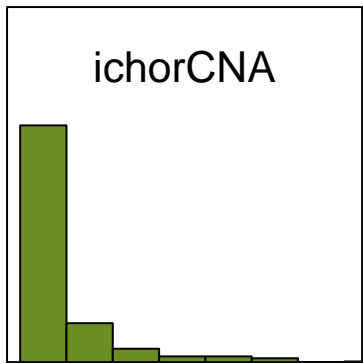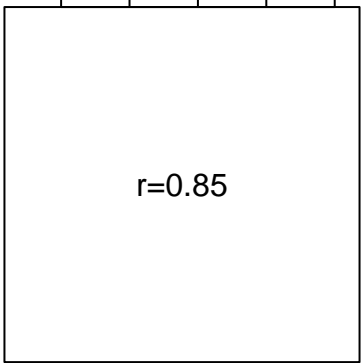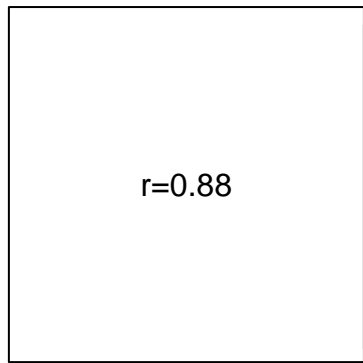

0 10 20 30 40 50 60 70

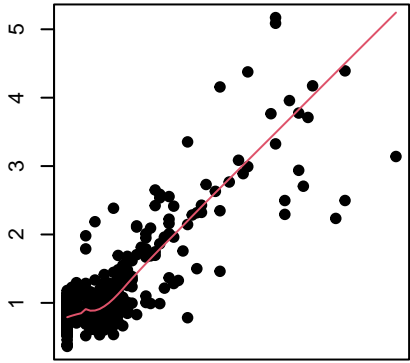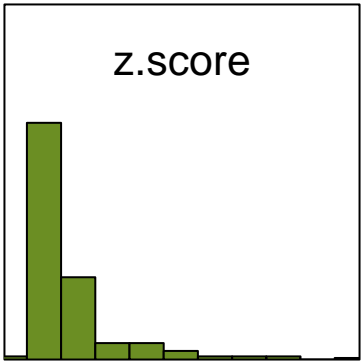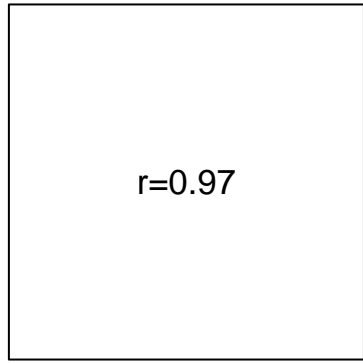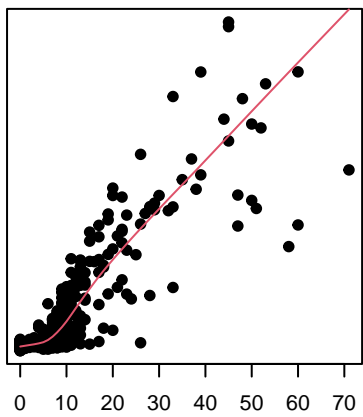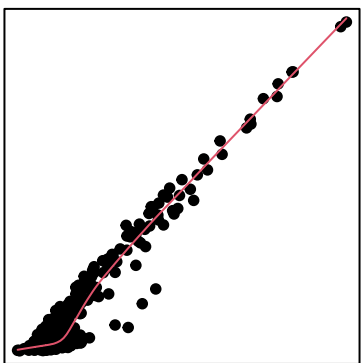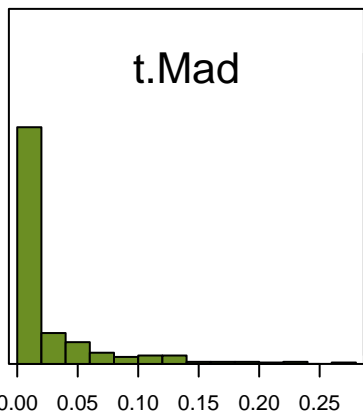

0.00 0.05 0.10 0.15 0.20 0.25

Supplement: Supplementary file 1 — Fig. S1. Alluvial plot showing selection of plasma samples for each analysis in the study. Fig. S2. Comparison of different scores to measure tumour fraction in ctDNA. Fig. S3. Identification of a threshold for the ichorCNA score using a spline term and segmented linear regression. Fig. S4. Overall survival in DETECT and Antwerp data. Fig. S5. Comparison of CA15‐3 and ichorCNA scores to estimate tumour fraction in 66 patients. Fig. S6. Discrepant results of ichorCNA measured with sWGS, mutant VAF measured with NGTAS and CA15‐3. Fig. S7. Prediction probabilities of progressive disease produced by BAY‐ML compared to the outcome of the CT scan, stratified according to the number of plasma samples available. [file MOL2-19-3518-s007.zip › FigureS2.pdf]

**a)**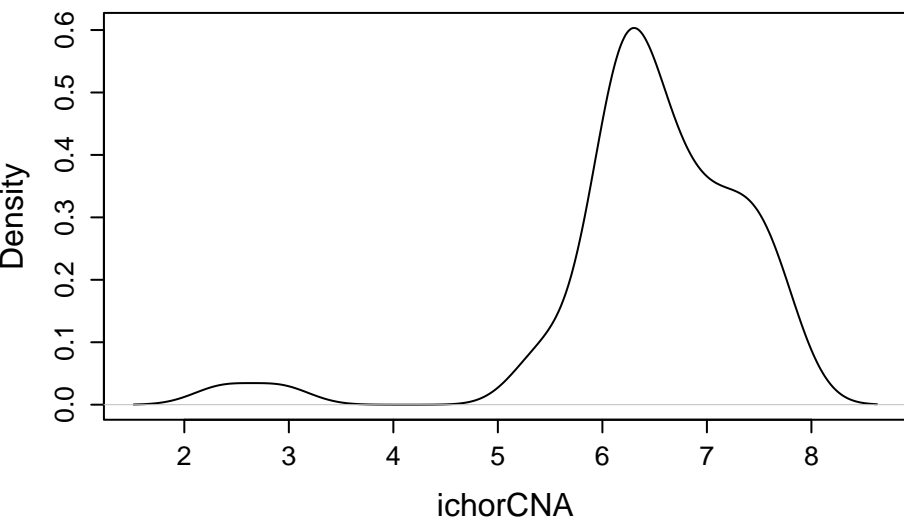**b)**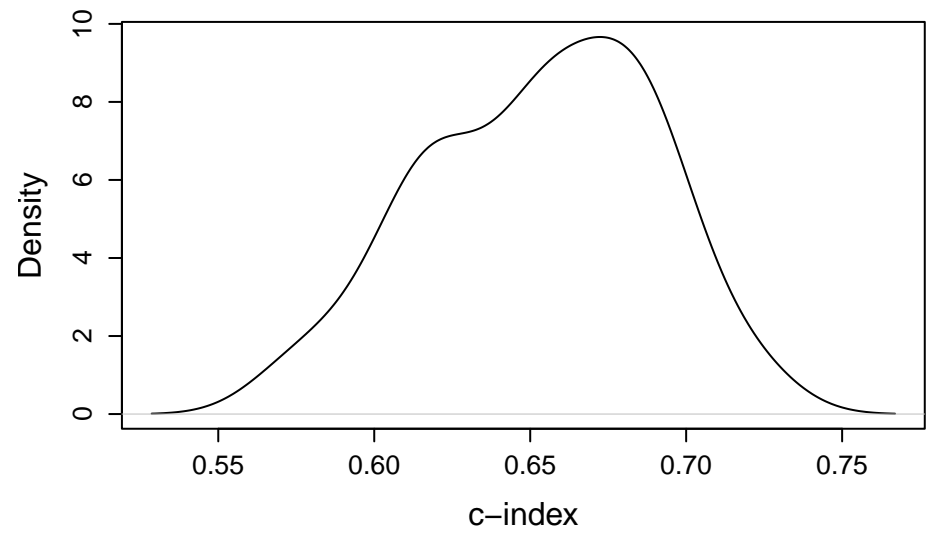**c)**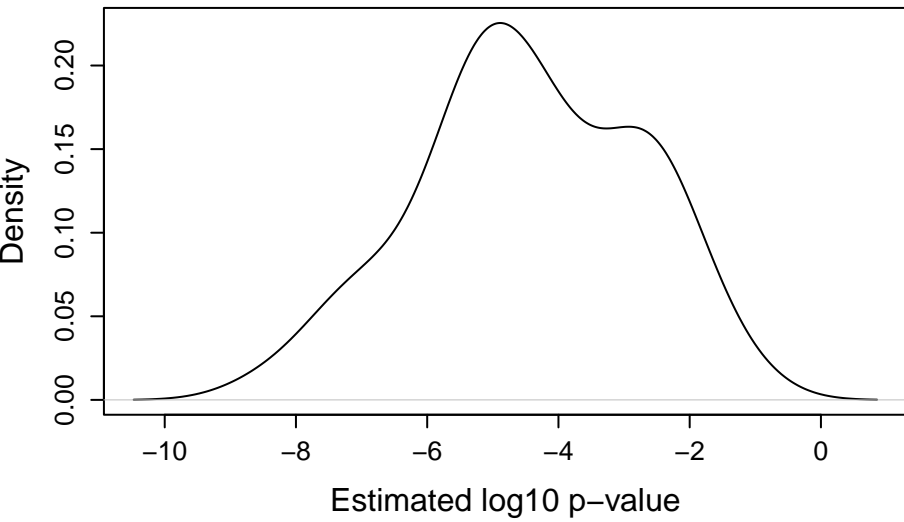**d)**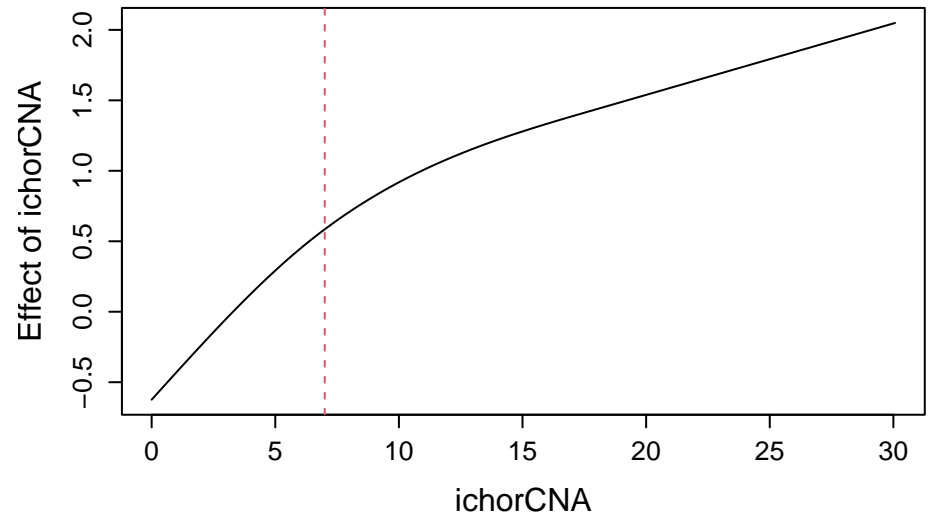**d) ER+/Her2-**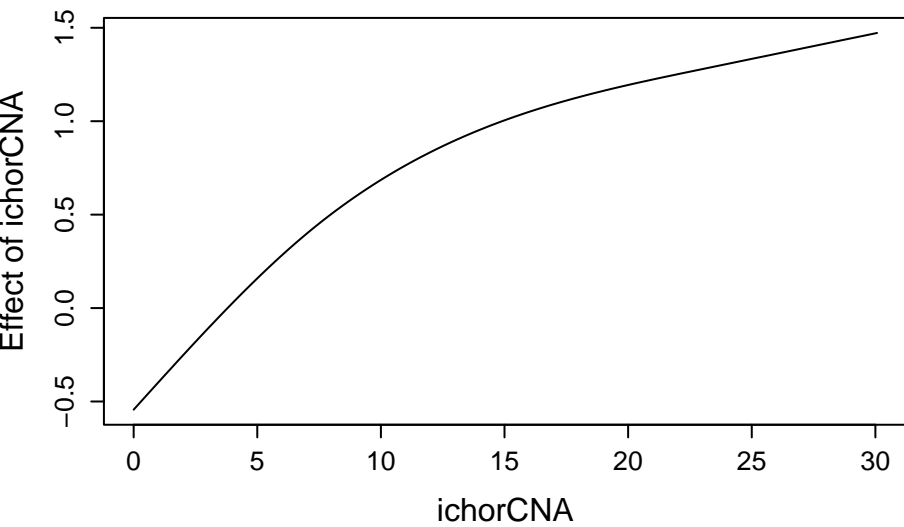**e) Her2-**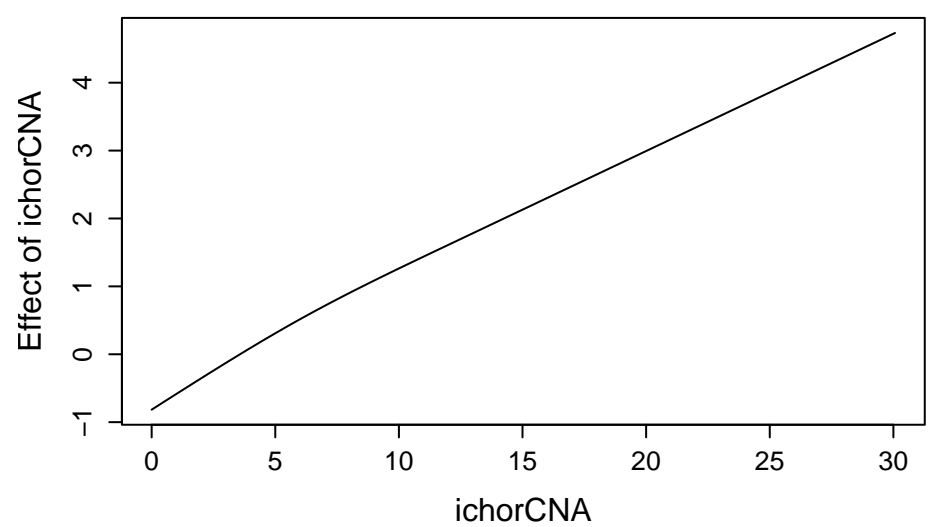

Supplement: Supplementary file 1 — Fig. S1. Alluvial plot showing selection of plasma samples for each analysis in the study. Fig. S2. Comparison of different scores to measure tumour fraction in ctDNA. Fig. S3. Identification of a threshold for the ichorCNA score using a spline term and segmented linear regression. Fig. S4. Overall survival in DETECT and Antwerp data. Fig. S5. Comparison of CA15‐3 and ichorCNA scores to estimate tumour fraction in 66 patients. Fig. S6. Discrepant results of ichorCNA measured with sWGS, mutant VAF measured with NGTAS and CA15‐3. Fig. S7. Prediction probabilities of progressive disease produced by BAY‐ML compared to the outcome of the CT scan, stratified according to the number of plasma samples available. [file MOL2-19-3518-s007.zip › FigureS3.pdf]

a)

ER+/Her2-  
(n=81)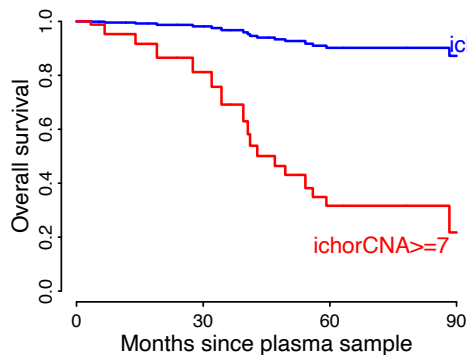Her2+  
(n=56)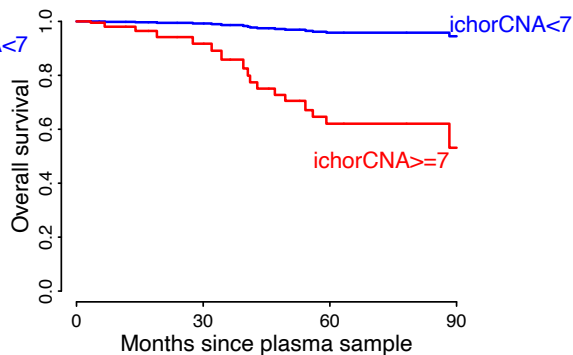

b)

ER+/Her2-  
(n=43)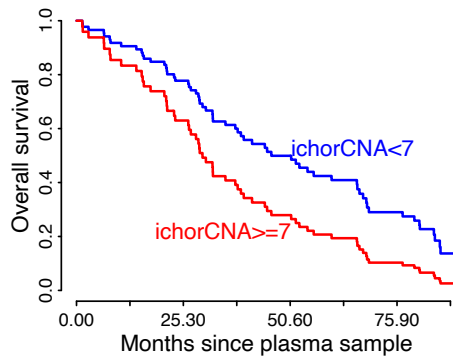Her2+  
(n=26)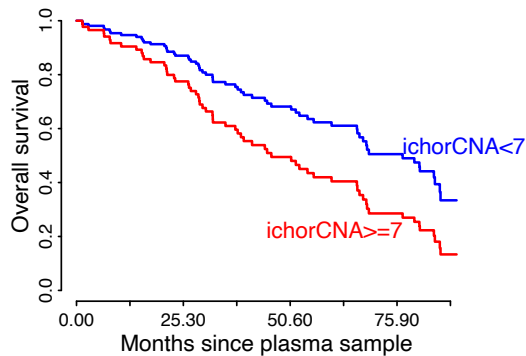TNBC  
(n=11)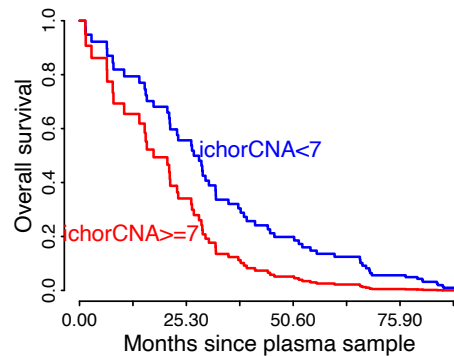

Supplement: Supplementary file 1 — Fig. S1. Alluvial plot showing selection of plasma samples for each analysis in the study. Fig. S2. Comparison of different scores to measure tumour fraction in ctDNA. Fig. S3. Identification of a threshold for the ichorCNA score using a spline term and segmented linear regression. Fig. S4. Overall survival in DETECT and Antwerp data. Fig. S5. Comparison of CA15‐3 and ichorCNA scores to estimate tumour fraction in 66 patients. Fig. S6. Discrepant results of ichorCNA measured with sWGS, mutant VAF measured with NGTAS and CA15‐3. Fig. S7. Prediction probabilities of progressive disease produced by BAY‐ML compared to the outcome of the CT scan, stratified according to the number of plasma samples available. [file MOL2-19-3518-s007.zip › FigureS4.pdf]

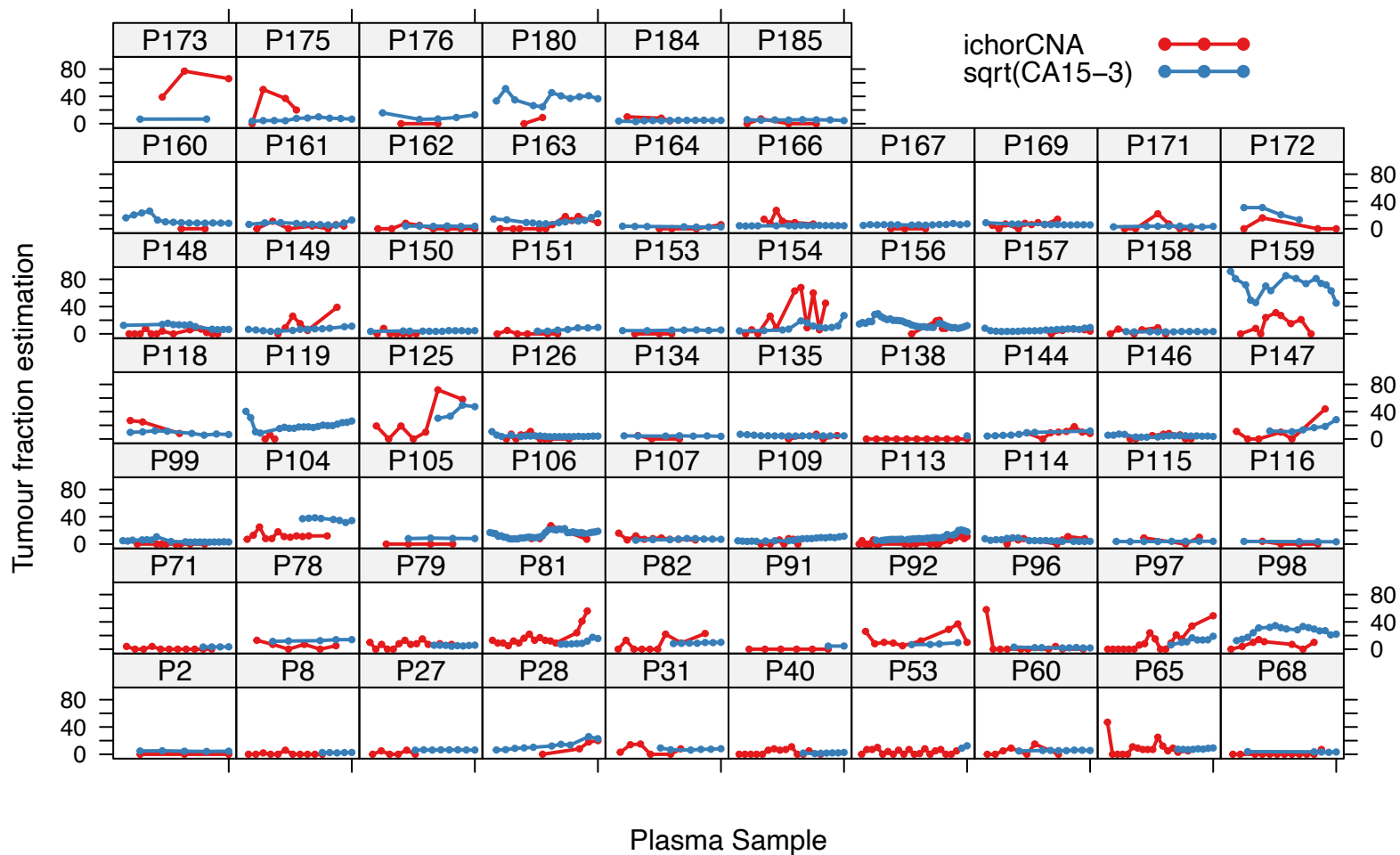

Supplement: Supplementary file 1 — Fig. S1. Alluvial plot showing selection of plasma samples for each analysis in the study. Fig. S2. Comparison of different scores to measure tumour fraction in ctDNA. Fig. S3. Identification of a threshold for the ichorCNA score using a spline term and segmented linear regression. Fig. S4. Overall survival in DETECT and Antwerp data. Fig. S5. Comparison of CA15‐3 and ichorCNA scores to estimate tumour fraction in 66 patients. Fig. S6. Discrepant results of ichorCNA measured with sWGS, mutant VAF measured with NGTAS and CA15‐3. Fig. S7. Prediction probabilities of progressive disease produced by BAY‐ML compared to the outcome of the CT scan, stratified according to the number of plasma samples available. [file MOL2-19-3518-s007.zip › FigureS5.pdf]

## Her2+

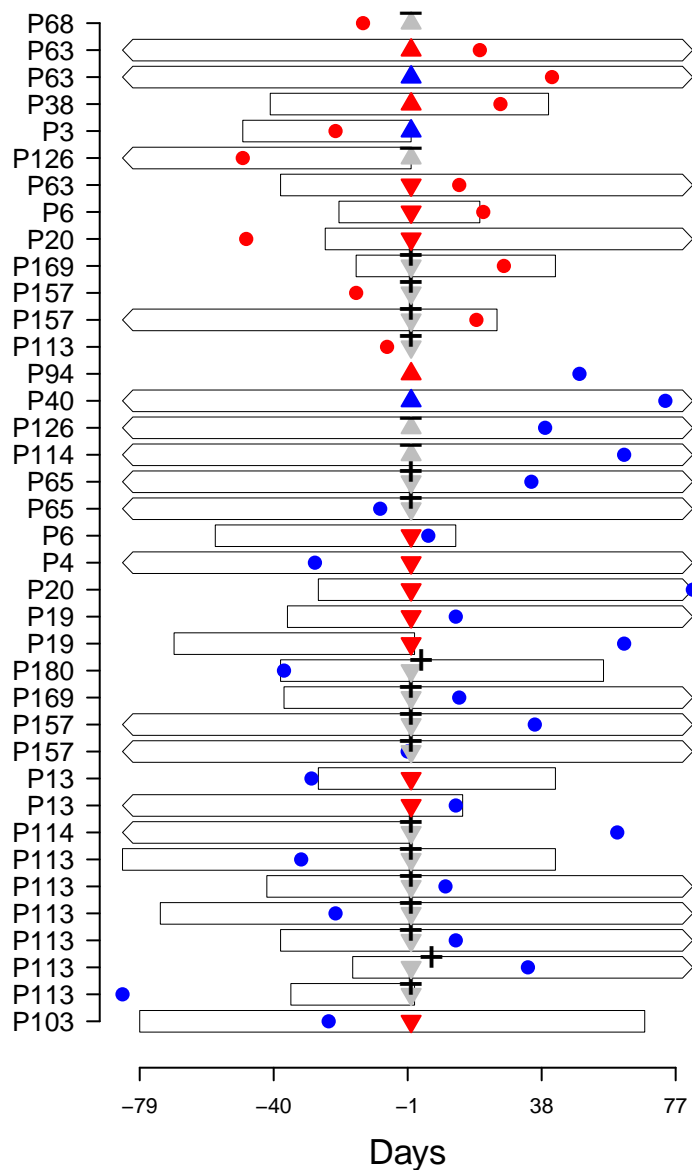

## ER+/Her2-

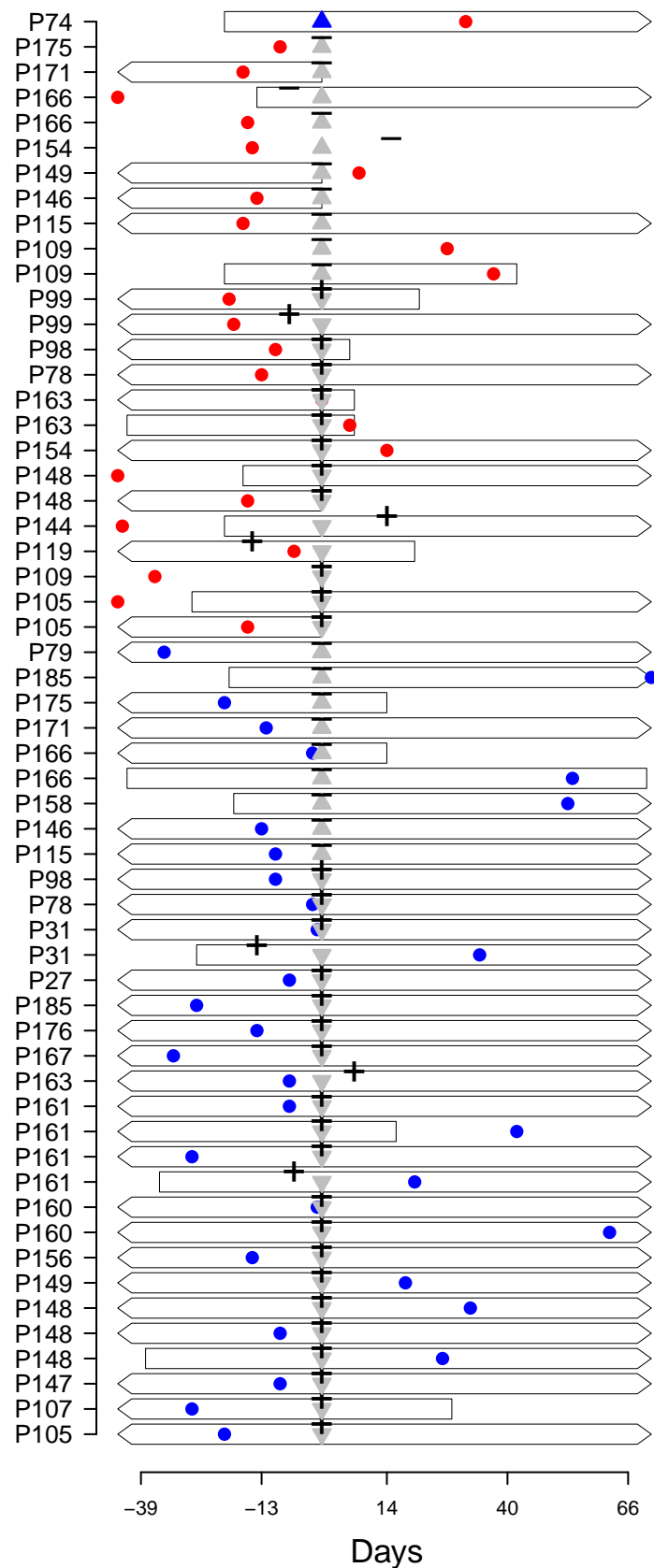

## ER-/Her2-

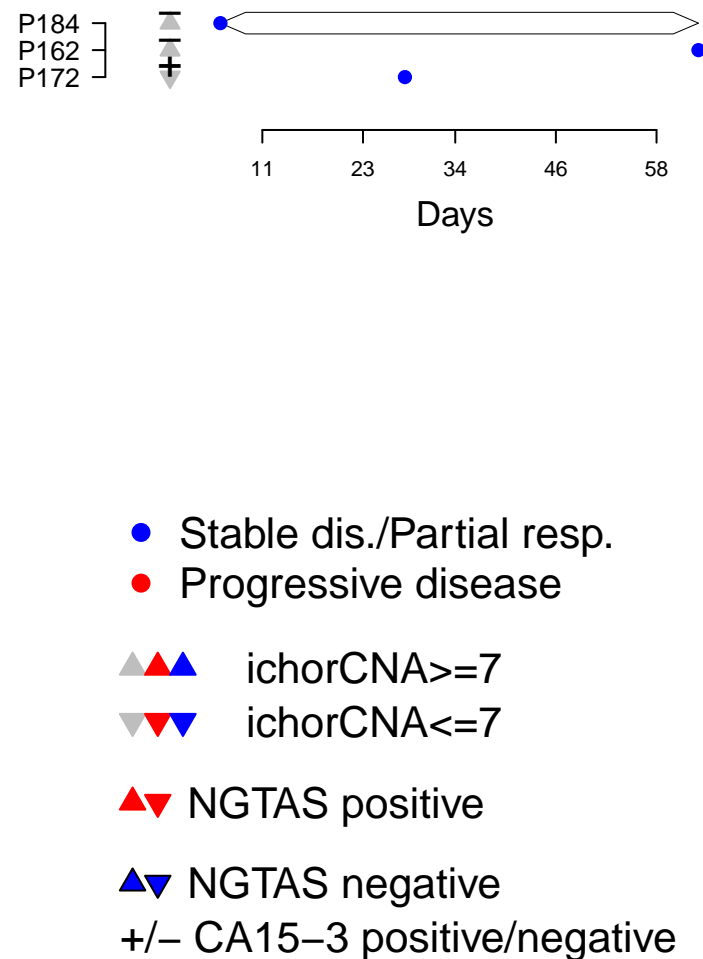

Supplement: Supplementary file 1 — Fig. S1. Alluvial plot showing selection of plasma samples for each analysis in the study. Fig. S2. Comparison of different scores to measure tumour fraction in ctDNA. Fig. S3. Identification of a threshold for the ichorCNA score using a spline term and segmented linear regression. Fig. S4. Overall survival in DETECT and Antwerp data. Fig. S5. Comparison of CA15‐3 and ichorCNA scores to estimate tumour fraction in 66 patients. Fig. S6. Discrepant results of ichorCNA measured with sWGS, mutant VAF measured with NGTAS and CA15‐3. Fig. S7. Prediction probabilities of progressive disease produced by BAY‐ML compared to the outcome of the CT scan, stratified according to the number of plasma samples available. [file MOL2-19-3518-s007.zip › FigureS6.pdf]

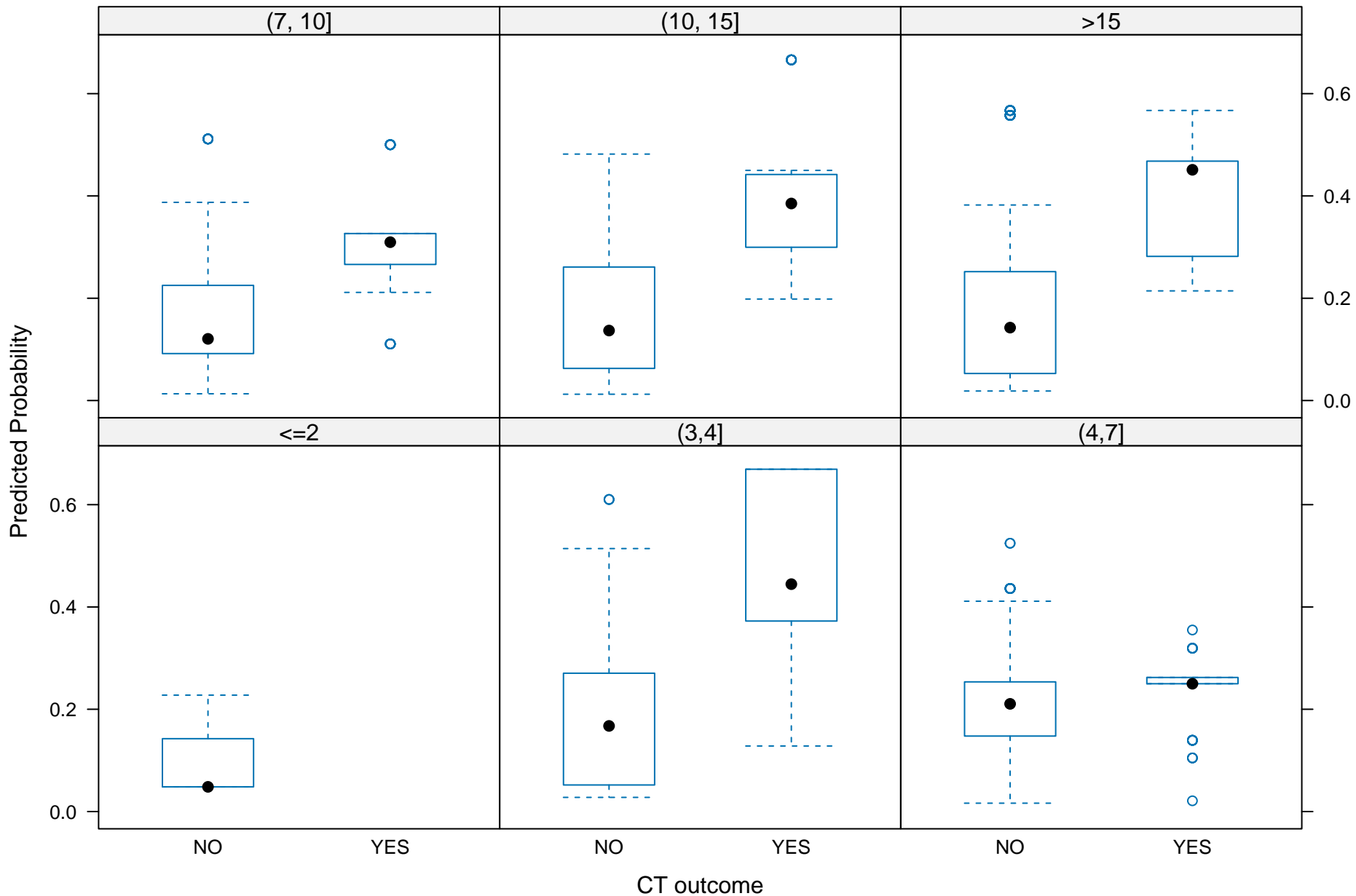

Supplement: Supplementary file 1 — Fig. S1. Alluvial plot showing selection of plasma samples for each analysis in the study. Fig. S2. Comparison of different scores to measure tumour fraction in ctDNA. Fig. S3. Identification of a threshold for the ichorCNA score using a spline term and segmented linear regression. Fig. S4. Overall survival in DETECT and Antwerp data. Fig. S5. Comparison of CA15‐3 and ichorCNA scores to estimate tumour fraction in 66 patients. Fig. S6. Discrepant results of ichorCNA measured with sWGS, mutant VAF measured with NGTAS and CA15‐3. Fig. S7. Prediction probabilities of progressive disease produced by BAY‐ML compared to the outcome of the CT scan, stratified according to the number of plasma samples available. [file MOL2-19-3518-s007.zip › FigureS7.pdf]
