# Supplementary material for: A large‐scale retrospective study in metastatic breast cancer patients using circulating tumour DNA and machine learning to predict treatment outcome and progression‐free survival
Source: Mol Oncol. 2025 Apr 15;19(12):3518–34. doi: 10.1002/1878-0261.70015 (PMC12688166; doi:10.1002/1878-0261.70015)
Supplement: Supplementary file 2 — Table S1. Parameter estimates in BAY‐ML. [file MOL2-19-3518-s008.docx]

**Table S1: Parameter estimates in the model**

Parameter estimates 1st stage model. l-95% CI and u-95% CI correspond to lower and upper 95% credible intervals.

|  |  |
| --- | --- |
| (Intercept) | 0.25 |
|  | [0.01, 0.48] |
| Time | 0.02 |
|  | [0.00, 0.04] |
| ER status | -0.05 |
|  | [-0.13, 0.03] |
| Her2 status | -0.02 |
|  | [-0.06, 0.03] |
| Capecitabine/5FU | -0.02 |
|  | [-0.18, 0.14] |
| Carboplatin/Gemcitabine | 0.04 |
|  | [-0.14, 0.21] |
| Docetaxel/Paclitaxel | -0.03 |
|  | [-0.20, 0.13] |
| EFC/CMF | 0.04 |
|  | [-0.19, 0.26] |
| Epirubicin | 0.04 |
|  | [-0.13, 0.20] |
| Eribulin | 0.04 |
|  | [-0.14, 0.22] |
| Exemestane | 0.06 |
|  | [-0.11, 0.22] |
| Exemestane/Everolimus | -0.10 |
|  | [-0.32, 0.12] |
| Fulvestrant | 0.10 |
|  | [-0.08, 0.28] |
| Lapatinib | -0.06 |
|  | [-0.31, 0.17] |
| Letrozole | -0.01 |
|  | [-0.18, 0.15] |
| Megestrol | 0.17 |
|  | [-0.16, 0.51] |
| Off Treatment | 0.15 |
|  | [-0.01, 0.31] |
| Tamoxifen | 0.00 |
|  | [-0.17, 0.17] |
| TDMM1 | 0.00 |
|  | [-0.17, 0.16] |
| Trastuzumab | 0.04 |
|  | [-0.13, 0.21] |
| Trastuzumab/Pertuzumab | 0.05 |
|  | [-0.13, 0.22] |
| Trial Drug | -0.04 |
|  | [-0.24, 0.16] |
| Vinorelbine | 0.11 |
|  | [-0.07, 0.28] |
| Treatment duration | 0.00 |
|  | [-0.01, 0.02] |
| $\sigma_{11}$ | 0.10 |
|  | [0.07, 0.12] |
| $\sigma_{12}$ | 0.05 |
|  | [0.03, 0.07] |
| $\rho$ | 0.43 |
|  | [-0.17, 0.94] |

Parameter estimates 2nd stage model. *γ*_1_ and *γ*_2_ are two elements of the *γ* vector.

|  |  |
| --- | --- |
| (Intercept) | -2.69 |
|  | [-6.85, 0.91] |
| time | 0.31 |
|  | [0.13, 0.50] |
| ER status | -0.87 |
|  | [-2.00, 0.18] |
| Her2 status | -0.30 |
|  | [-0.91, 0.29] |
| Capecitabine/5FU | 2.99 |
|  | [0.27, 6.60] |
| Carboplatin/Gemcitabine | 2.45 |
|  | [-0.53, 6.17] |
| Docetaxel/Paclitaxel | 1.19 |
|  | [-1.69, 4.87] |
| EFC/CMF | 2.64 |
|  | [-1.76, 7.14] |
| Epirubicin | 2.75 |
|  | [-0.05, 6.40] |
| Eribulin | 2.71 |
|  | [-0.39, 6.52] |
| Exemestane | 3.34 |
|  | [0.54, 7.02] |
| Exemestane/Everolimus | 2.60 |
|  | [-1.77, 7.08] |
| Fulvestrant | 2.80 |
|  | [-0.12, 6.51] |
| Lapatinib | -74.61 |
|  | [-218.10, 1.02] |
| Letrozole | 2.78 |
|  | [0.05, 6.40] |
| Megestrol | -76.47 |
|  | [-220.57, 0.26] |
| Off Treatment | 4.42 |
|  | [1.65, 8.07] |
| Tamoxifen | 3.92 |
|  | [1.10, 7.62] |
| TDMM1 | 2.74 |
|  | [0.01, 6.33] |
| Trastuzumab | 3.30 |
|  | [0.45, 7.01] |
| Trastuzumab/Pertuzumab | 2.79 |
|  | [-0.07, 6.49] |
| Trial Drug | 3.41 |
|  | [0.01, 7.45] |
| Vinorelbine | 3.90 |
|  | [0.92, 7.66] |
| $\gamma_{1}$ | 8.06 |
|  | [1.46, 15.30] |
| $\gamma_{2}$ | 4.38 |
|  | [-10.55, 19.13] |
| $\sigma_{u}$ | 0.88 |
|  | [0.44, 1.35] |
|  | |
